# Differences in glycosyltransferase family 61 accompany variation in seed coat mucilage composition in *Plantago* spp.

**DOI:** 10.1093/jxb/erw424

**Published:** 2016-11-17

**Authors:** Jana L. Phan, Matthew R. Tucker, Shi Fang Khor, Neil Shirley, Jelle Lahnstein, Cherie Beahan, Antony Bacic, Rachel A. Burton

**Affiliations:** ^1^Australian Research Council Centre of Excellence in Plant Cell Walls, School of Agriculture, Food and Wine, University of Adelaide, Waite Campus, Urrbrae, SA 5064, Australia; ^2^Australian Research Council Centre of Excellence in Plant Cell Walls, School of Botany, University of Melbourne, Parkville Campus, VIC 3010, Australia

**Keywords:** Glycosyltransferase family 61, heteroxylan, *Plantago*, plant cell walls, polysaccharide biosynthesis, seed mucilage.

## Abstract

Xylans are the most abundant non-cellulosic polysaccharide found in plant cell walls. A diverse range of xylan structures influence tissue function during growth and development. Despite the abundance of xylans in nature, details of the genes and biochemical pathways controlling their biosynthesis are lacking. In this study we have utilized natural variation within the *Plantago* genus to examine variation in heteroxylan composition and structure in seed coat mucilage. Compositional assays were combined with analysis of the glycosyltransferase family 61 (GT61) family during seed coat development, with the aim of identifying GT61 sequences participating in xylan backbone substitution. The results reveal natural variation in heteroxylan content and structure, particularly in *P. ovata* and *P. cunninghamii*, species which show a similar amount of heteroxylan but different backbone substitution profiles. Analysis of the GT61 family identified specific sequences co-expressed with *IRREGULAR XYLEM 10* genes, which encode putative xylan synthases, revealing a close temporal association between xylan synthesis and substitution. Moreover, in *P. ovata*, several abundant GT61 sequences appear to lack orthologues in *P. cunninghamii.* Our results indicate that natural variation in *Plantago* species can be exploited to reveal novel details of seed coat development and polysaccharide biosynthetic pathways.

## Highlight

Seed coat mucilage composition and heteroxylan structure varies between different *Plantago* species and is accompanied by differences in glycosyltransferase family 61 (GT61) copy number and transcript abundance.

## Introduction

During plant development, the seed coat of myxospermous species differentiates to produce specialized cells containing mucilage, a polysaccharide-rich substance (Hyde, 1970). Upon contact with aqueous environments, the mucilage-producing cells rupture and mucilage is extruded, possibly aiding in seed dispersal and hydration during germination (Yang *et al.*, 2012). Although the majority of plants found in arid sandy soils are myxospermous (Deng *et al.*, 2012), the precise function of mucilage remains unknown, since mutants that lack the ability to extrude mucilage are still viable (Penfield *et al.*, 2001; Huang *et al.*, 2011). In addition, seed mucilage composition varies considerably across different genera. Arabidopsis is a well-documented myxospermous species that produces seed mucilage rich in rhamnogalacturonan I with smaller amounts of homogalacturonan, cellulose, xyloglucan, xylan, arabinan and galactan (Haughn and Western, 2012; Voiniciuc *et al.*, 2015*b*, 2016; Hu *et al.*, 2016). Flax (*Linum usitatissimum*) produces mucilage containing rhamnogalacturonan I and arabinoxylan (Naran *et al.*, 2008), while pysllium (*Plantago ovata*) mucilage is predominantly composed of a complex heteroxylan (Fischer *et al.*, 2004; Saghir *et al.*, 2008). Although reasons for diversity in mucilage composition are unclear, the abundance and accessibility of complex polysaccharide mixtures in mucilage make it an attractive system to study polysaccharide biosynthesis and modification. In recent times, this has been shown by the development of *P. ovata* as a model system to study xylan biosynthesis (Jensen *et al.*, 2011, 2013, 2014).

The backbone of xylan polysaccharides consists of β-1,4-linked xylopyranosyl residues. Structural heterogeneity is achieved through addition of various monosaccharides, methyl and acetyl groups onto the backbone, forming single, double or longer sidechains. These sidechains make for a more complex polysaccharide that can interact and form linkages with neighbouring polysaccharides, creating the framework that is essential for plant cell wall integrity (Cosgrove, 2005). Xylan biosynthesis occurs in the Golgi apparatus (Rennie and Scheller, 2014) where enzymes including glycosyltransferases (GTs) are required to catalyse glycosidic linkages between each monosaccharide moiety. Although a comprehensive molecular understanding is lacking, by analysing xylan deficient mutants, candidate genes involved in xylan biosynthesis have been identified (for review see Rennie & Scheller, 2014). In eudicots, GT43 (IRX9, IRX9-L, IRX14 and IRX14-L) and GT47 (IRX10 and IRX10-L) enzymes have been implicated in synthesis of the backbone. Recent investigations surrounding IRX10-L indicate that GT47 is likely to be an essential catalytic component required for the addition of UDP-xylose moieties onto a growing β-1,4-xylan backbone, leading to its designation as XYLAN SYNTHASE-1 (Jensen *et al.*, 2014; Urbanowicz *et al.*, 2014). Specific GTs that catalyse the addition of variable sidechains onto the backbone are also of interest, particularly from an applied perspective, because they play an important role in polysaccharide solubility and viscosity. In Arabidopsis, the GUX GT8 proteins function as glucuronyltransferases (Lee *et al.*, 2012) and in some cereal species, GT61 family members have been implicated in β-1,2-xylosyltransferase and/or α-1,3-arabinoxyltransferase activity (Anders *et al.*, 2012; Chiniquy *et al.*, 2012). A role for the GT61 family in seed mucilage has recently been shown in Arabidopsis, where the *MUCI21* gene influences mucilage organization, possibly through modification of small amounts of xylan (Voiniciuc *et al.*, 2015*a*; Ralet *et al.*, 2016). In addition, Jensen *et al.* (2013) identified an abundance of GT61 sequences in the mucilage-producing cells of *P. ovata*.

In Arabidopsis, forward and reverse genetic approaches have been used to identify genes involved in seed mucilage polysaccharide biosynthesis and release (Haughn and Western, 2012). Other approaches have made use of natural variation between different accessions to identify quantitative trait loci (QTLs) and novel genes (Saez-Aguayo *et al.*, 2013). In *P. ovata*, Jensen *et al.* (2013, 2014) used a reverse genetics approach to identify IRX10 homologues and other GTs by generating transcript profiles from the developing integument. The *Plantago* genus contains almost 500 species (Mohsenzadeh *et al.*, 2008), but the natural variation present between these species, specifically relating to seed mucilage heteroxylan, has yet to be investigated. Here we present our analysis of seven *Plantago* species and provide clear evidence of compositional and structural variation in mucilage polysaccharide content, particularly for heteroxylan. Transcriptomic profiles of integument tissue from *P. ovata* and *P. cunninghamii*, which contain similar amounts of xylan but with a different degree of backbone substitution, allowed for a detailed analysis of sequences predicted to be involved in heteroxylan biosynthesis. Examination of the GT61 family indicated differences in mucilage heteroxylan structure, particularly in *P. ovata*, are accompanied by changes in GT61 gene copy number and transcript accumulation patterns. The results suggest that the diverse gene pool present in the *Plantago* genus can be used to identify novel genes and enzymatic activities involved in xylan biosynthesis.

## Materials and methods

### Plant growth conditions

*Plantago* seeds were obtained from sources listed in Supplementary Table S1 at *JXB* online. Seeds were sterilized in 50% bleach and 50% ethanol for 1 min and five 1 min washes of milliQ water (with the exception of *P. major*, which was sprinkled directly onto soil). *P. ovata*, *P. coronopus*, *P. debilis*, and *P. lanceolata* were germinated on agar plates (0.5× Murashige and Skoog basal medium, 1% sucrose at pH 5.7) in growth chambers with 16 h photoperiod and day/night temperatures of 22 °C/18 °C. *P. cunninghamii* and *P. varia* were germinated on filter paper at room temperature. All seedlings were transplanted into a coco-peat soil mixture around 10 d post-germination. Plants were grown in a glasshouse with day/night temperatures of 23 °C/18 °C from December through to March with no supplemental lighting (Adelaide, Australia).

### Seed mucilage extraction

Seed mucilage was extracted using a method adapted from Balke and Diosady (2000). Whole seeds were obtained from a number of different plants and pooled for extraction: 1 g was hydrated in 25 ml water and stirred at 80 °C for 90 min. Hot water extraction was sufficient to remove all seed mucilage from most species (see Supplementary Fig. S1 at *JXB* online), while 0.2 M KOH was required in the case of *P. ovata*; however, monosaccharide analysis revealed insignificant differences in composition (J. Phan, L. Yu, unpublished data). Liberated mucilage was extracted using a sieve of fine tulle (Crystal Tulle, Spotlight, Australia). Samples were snap frozen in liquid nitrogen, freeze dried for 48 h, ground over liquid nitrogen and stored in an air-tight container at room temperature. Before analysis, mucilage samples were separated into two fractions, alcohol soluble and alcohol insoluble, using ethanol precipitation (Pettolino *et al.*, 2012).

### Microscopy

#### Light microscopy

Ruthenium red staining followed the protocol from Arsovski *et al.* (2009) with modifications excluding pre-hydration and imbibition for 10 min. Imaging was conducted using a Zeiss Stemi 2000-C dissecting microscope with an attached AxioCam ERc 5s camera. For calcofluor white staining, mature seeds were imbibed in a solution of 0.002% (w/v) calcofluor white (Sigma-Aldrich, F3543) with 0.01% Triton X-100 (Sigma-Aldrich, T8532) in 100 ml Tris (pH 8.0) for 10 min and imaged using a Leica AS LMD laser dissection microscope with an attached DFC 480 camera. To confirm developmental stages, seeds were cleared as per Tucker *et al.* (2012) using Hoyer’s light solution (Anderson, 1954) and observed using differential interference contrast (DIC) and Nomarski optics on a Zeiss M2 Axio imager. To observe anatomical details of seed coat development, staged seed samples were fixed in 0.25% glutaraldehyde, 4% paraformaldehyde and 4% sucrose in phosphate-buffered saline (PBS; pH 7.2), embedded in LR-White resin and sectioned to 1.0 μm before staining in 0.01% toluidine blue in 0.1% sodium tetraborate as per Aditya *et al*. (2015).

#### Scanning electron microscopy

Whole mature seeds were coated with a thin layer of carbon prior to viewing and imaging using a Philips XL20 Scanning Electron Microscope.

#### *Whole-mount and thin-section immunolabelling of extruded* Plantago *seed mucilage*

For localization of heteroxylan a protocol was adapted from Willats *et al.* (2001). Mature seeds were hydrated in 1×PBS for 30 min with gentle shaking and incubated for 90 min in a 10-fold dilution of primary antibody, LM11 (McCartney *et al.*, 2005). Samples were washed in 1×PBS (5 × 1 min) and incubated for 90 min in a 100-fold dilution of goat anti-rat IgG conjugated with Alexa Fluor^®^ 488 (Invitrogen, USA, A1100), washed as above and counterstained with 0.2 µg ml^–1^ propidium iodide (Sigma-Aldrich, P417). Whole seeds were mounted in 1×PBS and imaged using a Leica AS LMD laser dissection microscope with attached DFC 480 camera.

For double labelling with CBM3a, samples were incubated with a 10-fold dilution of primary antibody LM11 and 100-fold dilution of secondary antibody goat anti-rat IgM conjugated with DyLight 550 followed by a 500-fold dilution of CBM3a and a 300-fold dilution of mouse anti-His antibody. The His probe was labelled with goat anti-mouse IgG conjugated with Alexa Fluor 488. All antibodies were applied for 60 min with gentle shaking and samples washed in 1× PBS (5 × 1 min) between each incubation.

For immunolabelling of thin sections, the protocol of Burton *et al.* (2011) was followed. Transmission electron microscopy was carried out using a Philips CM100 microscope (Adelaide Microscopy).

### Monosaccharide profiling

Monosaccharide profiles were determined using reverse phase high performance liquid chromatography (RP-HPLC) of 1-phenyl-3-methyl-5-pyrazoline (PMP) derivatives as per Burton *et al.* (2011).

### Oligosaccharide mapping

#### Pre-treatment

Ground *Plantago* seed mucilage was treated with 0.1 M trifluoroacetic acid (TFA) (Agilent Technologies, USA, G2007A) at 85 °C for 60 min with gentle shaking and precipitated with a series of 70% ethanol washes.

#### Xylanase digest

Fresh and pre-treated samples were digested with 4.875 U of endo-1,4-β-D-xylanase (Megazyme, E-XYNBCM, Ireland) overnight at room temperature. Digests were collected via centrifugation and incubated at 100 °C for 10 min.

#### Chromatography

Samples were diluted (1/20) and analysed using RP-HPLC on a Dionex IC5-5000 ICS LC with a CarboPac PA200 3 × 250 mm + guard column at 35 °C and auto-sampler at 10 °C. Pulse amperometric detection (PAD) of samples was recorded at 2 Hz, with the detector kept at 20 °C. Eluents consisted of 0.1 M sodium hydroxide (A) and 0.1 M sodium hydroxide and 1.0 M sodium acetate (B). Column flow rate was 0.5 ml min^–1^ for a total run time of 12 min. The initiating gradient condition was 99% A; at 9 min this decreased to 85%, at 10 min to 100% and at 12 min the gradient was returned to starting conditions.

### Methylation linkage analysis

Seed mucilage was analysed as per Pettolino *et al.* (2012)

### Tissue harvest, RNA extraction and cDNA synthesis

Integument tissues were collected in triplicate from fruits at 1–16 d after pollination (DAP); leaf, bract and general tissues were harvested when plants were 2 months old. All fresh tissue was placed into liquid nitrogen and stored at –80 °C until required. Vascular bundles were removed from leaf tissue using a razor blade.

Total RNA was extracted using the Spectrum^TM^ Plant Total RNA kit (Sigma-Aldrich, USA). The Superscript^®^ III Reverse Transcriptase kit (Invitrogen, USA) was used to synthesize cDNA according to Burton *et al.* (2008). cDNAs from two to three biological replicates were generated for each stage of seed coat development.

### RNAseq analysis

Total RNA samples were submitted to the Australian Genome Research Facility Ltd (Melbourne, Australia) to generate RNA sequencing (RNAseq) libraries using the Illumina HiSeq platform. Raw sequence data were assembled using CLC Genomics Workbench v8.0 (CLC bio, Aarhus, Denmark). Contig sequences were BLASTed against the Arabidopsis database to assign annotations (https://www.Arabidopsis.org/tools/bulk/sequences/index.jsp). The largest open reading frames were extracted and translated proteins were analysed for functional motifs by searching for PFAM domains.

Illumina reads were trimmed then assembled into contig sequences and gene expression was calculated in RPKM.

*P. cunninghamii* SRA data are lodged at NCBI as SRP078232 and *P. ovata* data are lodged as SRP078381.

### Phylogenetic analysis

All bioinformatics analyses were conducted using Geneious^®^ Pro version 8.1.3 (http://www.geneious.com; Kearse *et al.*, 2012). Several *P. ovata* RNA contigs *encoding putative GT61, GT47 and CesA sequences* were identified based on sequences published in Jensen *et al.* (2013). Reciprocal best BLAST was used to identify a set of GT61 genes in *P. ovata* and *P. cunninghamii*. *P. ovata* GT61 homologues in *P. cunninghamii* were identified using a combination of Megablast/blastn and tblastx within Geneious. Results were ranked according to the lowest e-value. *P. cunninghamii* contigs ranked within the top three hits of tblastx (for each *P. ovata* GT61) were BLASTed against the *P. ovata* RNA-seq library to identify *P. cunninghamii* GT61s and *P. ovata* GT61s not previously identified.

Trees were constructed based on the predicted coding sequence of *P. ovata* and *P. cunninghamii* GT61s identified using BLAST. Multiple alignments of coding sequences were performed using the default setting of MUSCLE. An approximate maximum likelihood phylogenetic tree was constructed using the FastTree plugin (Price *et al.*, 2010) in Geneious Pro version 8.1.3. Coding sequences of GT61 genes in Arabidopsis were extracted from Phytozome^®^ (Goodstein *et al.*, 2012; http://www.phytozome.net/) using Pfam ID PF04577 published in Anders *et al.* (2012), as well as the GT61 gene entries in CAZy (Lombard *et al.*, 2014).

### Quantitative polymerase chain reaction

*IRX10*, *UXS*, *UAM* and *GT61* quantitative polymerase chain reaction (qPCR) primers were generated using Primer3^®^ version 0.4.0. (Untergasser *et al.*, 2012).

qPCR was conducted based on Burton *et al.* (2008); primers and PCR products are described in Supplementary Table S2, with the standard curve templates generated using HPLC (Trafford *et al.*, 2013). Two biological replicates of cDNAs from integument tissue, 1–16 DAP, were used. Normalization factors from control genes (see Supplementary Table S2 at *JXB* online) were determined according to Vandesompele *et al.* (2002) and Burton *et al.* (2004). Normalization factors were generated from the geometric means of three control genes (Vandesompele *et al.*, 2002; Burton *et al.*, 2004).

## Results

### *Plantago* species show natural variation in seed mucilage patterning

**Fig. 1.**
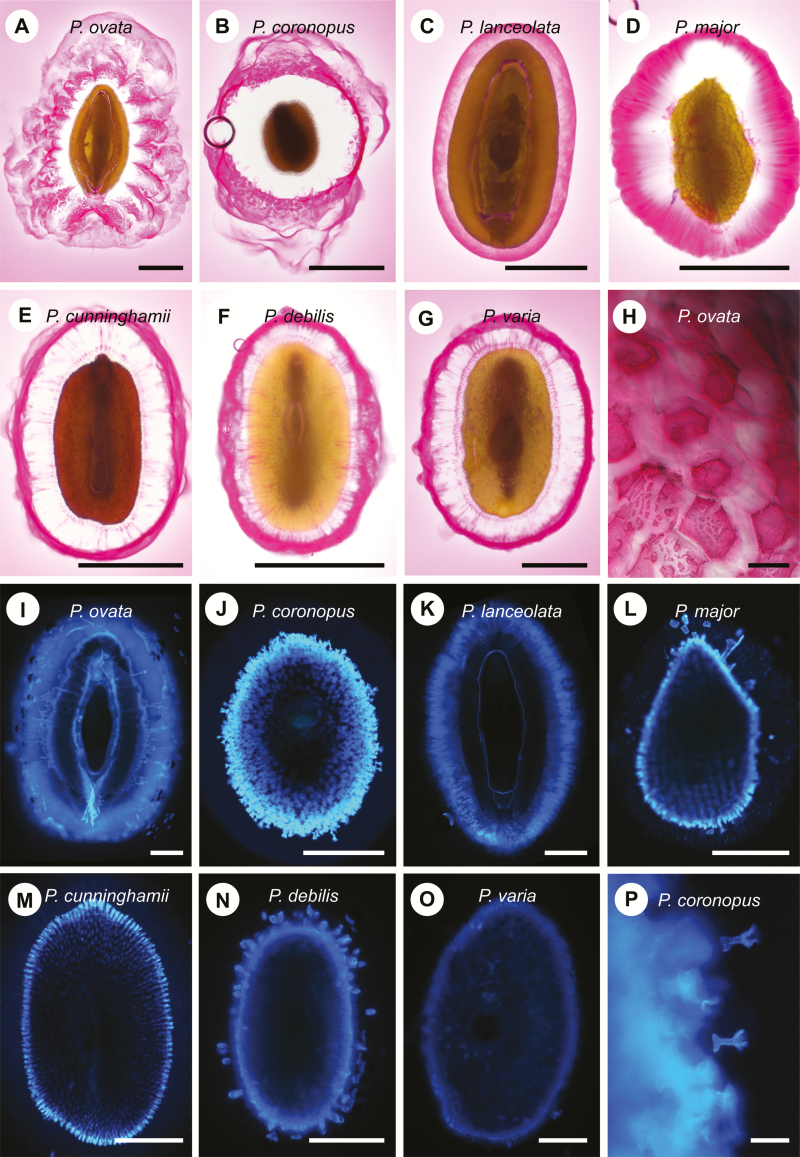
Histological stains reveal differences in mucilage release and composition in different *Plantago* species. (A–H), whole-mount seeds imbibed in ruthenium red; (I–P), seeds stained in calcofluor white. (H) Magnified view of mucilage morphology in *P. ovata*, showing the characteristic hexagonal shape of mucilage plates surrounded by unstained regions of polysaccharide. (P) Magnified view of seed coat ‘rockets’ ejecting from the *P. coronopus* seed coat. Bar A–G, I–O: 500 µm; H: 20 µm; P: 100 µm.

Histological analyses identified variations in polysaccharide composition and structure of the extruded seed mucilage in all species (Fig. 1). Acidic polysaccharides, including pectin, stain reddish-pink in ruthenium red. While all species showed strong mucilage staining, *P. ovata* showed an irregular ruthenium red staining pattern (Fig. 1A), comprising three distinct layers: a cloud-like outer layer, a middle layer that appeared to be concentrated in hexagonal platelets (Fig. 1H) and an inner unstained layer. *P. coronopus* produced a staining pattern characterized by a thick unstained inner layer, while the outer layer stained intensely with ‘swirls’ of mucilage diffusing in concentric rings (Fig. 1B). Mucilage extruded from *P. lanceolata* did not diffuse far from the seed coat (Fig. 1C), and formed a single homogeneous layer with clearly defined edges. Acidic components in the extruded mucilage of *P. major* stained to reveal a pattern that radiated from the seed with a gradient of staining intensity (Fig. 1D). *P. cunninghamii*, *P. debilis*, and *P. varia* (Fig. 1E–G) produced similar ruthenium red staining patterns with heavily stained outer layers and pink striations through a partly transparent inner layer.

Calcofluor white staining, which detects β-glucans such as cellulose (Harrington and Hageage, 2003), was evident in the seed coat (Fig. 1I–P) but weak in the extruded mucilage of most species. Strong staining was detected for *P. coronopus*, *P. major* and *P. debilis* (Fig. 1J, L, N), but appeared to correspond to entire cells ejected from the seed coat rather than individual strands of β-glucan (Fig. 1P).

### Plantago seed mucilage composition varies between species

Monosaccharide analysis of hot-water extracted seed mucilage (Table 1 and Supplementary Table S3) indicated xylose and arabinose were abundant in all species, both in total mucilage extract and alcohol insoluble fractions. The relative abundance of each sugar varied considerably, *P. ovata* mucilage containing the most xylose and *P. coronopus* containing the least (~62% w/w and ~16% w/w respectively). By contrast, *P. varia* mucilage contained the highest amount of arabinose (~19% w/w) and *P. lanceolata* the least (~5% w/w). Other monosaccharides varied between species. For example, glucuronic acid was barely detected or absent in most species except *P. major* (~1.5% w/w), while galactose was most abundant in *P. coronopus* (~8% w/w). Only glucose, galactose, rhamnose, and glucuronic acid monosaccharides were identified in the alcohol-soluble mucilage fractions, although these contributed less than 10% of total mucilage weight.

**Table 1.** *Monosaccharide analysis on different fractions of extruded seed mucilage from* Plantago *species* Values are means±SD in mol%; *n* = 3 technical replicates; SD, standard deviation; ND, not detected.

Species	Man	Rib	Rha	GlcAc	GalAc	Glu	Gal	Xyl	Ara
Whole mucilage									
*P. ovata*	2.0 ± 1.3	ND	2.3 ± 0.2	ND	2.6 ± 0.2	1.9 ± 0.1	2.0 ± 0.2	61.5 ± 5.8	17.2 ± 1.6
*P. coronopus*	2.2 ± 1.5	ND	4.5 ± 0.5	0.1 ± 0.1	2.7 ± 0.3	8.8 ± 1.1	8.2 ± 0.9	15.7 ± 1.5	8.8 ± 0.9
*P. cunninghamii*	1.5 ± 1.0	ND	5.3 ± 0.1	ND	2.3 ± 0.1	2.6 ± 0.1	1.2 ± 0.0	55.2 ± 1.2	16.2 ± 0.3
*P. debilis*	1.4 ± 0.9	ND	4.2 ± 0.3	ND	2.6 ± 0.3	4.7 ± 0.3	4.1 ± 0.3	41.3 ± 2.0	15.8 ± 0.9
*P. lanceolata*	1.7 ± 0.8	ND	2.6 ± 0.2	0.4 ± 0.3	2.8 ± 0.3	9.3 ± 0.6	5.2 ± 0.3	38.7 ± 2.0	4.7 ± 0.2
*P. major*	0.1 ± 0.0	0.2 ± 0.4	1.0 ± 0.2	1.5 ± 0.5	0.9 ± 0.2	12.0 ± 0.7	2.1 ± 0.1	36.8 ± 2.7	5.1 ± 0.4
*P. varia*	1.5 ± 0.9	ND	2.7 ± 0.5	0.1 ± 0.2	2.7 ± 0.4	7.3 ± 1.2	5.2 ± 0.9	41.2 ± 8.3	19.5 ± 3.8
Alcohol insoluble									
*P. ovata*	ND	ND	ND	ND	ND	3.4 ± 5.8	ND	54.5 ± 13.5	15.5 ± 3.1
*P. coronopus*	ND	ND	5.1 ± 1.5	ND	5.6 ± 2.0	3.8 ± 6.7	7.1 ± 1.3	24.7 ± 2.9	13.8 ± 1.8
*P. cunninghamii*	ND	ND	7.3 ± 1.7	3.3 ± 5.8	ND	3.3 ± 5.7	ND	60.9 ± 6.0	20.7 ± 2.8
*P. debilis*	ND	ND	2.7 ± 2.4	ND	ND	6.8 ± 5.6	3.1 ± 2.7	46.6 ± 1.7	19.6 ± 0.8
*P. lanceolata*	ND	ND	1.1 ± 1.9	3.6 ± 6.3	5.5 ± 2.0	3.8 ± 6.5	6.3 ± 1.2	48.9 ± 4.6	5.0 ± 1.2
*P. major*	ND	ND	ND	7.3 ± 4.0	ND	6.6 ± 6.4	1.3 ± 2.2	52.3 ± 2.4	8.3 ± 1.0
*P. varia*	ND	ND	ND	3.7 ± 6.4	ND	5.5 ± 9.6	1.4 ± 2.4	52.2 ± 0.9	24.0 ± 0.8
Alcohol soluble									
*P. ovata*	ND	ND	ND	11.3 ± 8.0	ND	21.8 ± 9.2	ND	ND	ND
*P. coronopus*	ND	ND	4.7 ± 1.2	4.5 ± 7.8	ND	23.9 ± 7.0	12.3 ± 2.3	ND	ND
*P. cunninghamii*	ND	ND	2.3 ± 2.0	5.6 ± 6.5	ND	29.9 ± 7.8	10.9 ± 2.2	ND	ND
*P. debilis*	ND	ND	2.4 ± 2.1	6.4 ± 7.5	ND	26.6 ± 7.3	10.1 ± 2.8	ND	ND
*P. lanceolata*	ND	ND	ND	6.5 ± 8.1	ND	29.7 ± 8.1	3.5 ± 601	ND	ND
*P. major*	ND	ND	ND	6.8 ± 8.4	ND	33.3 ± 7.2	6.8 ± 2.0	ND	ND
*P. varia*	ND	ND	1.2 ± 2.1	5.9 ± 7.2	ND	23.5 ± 7.8	9.9 ± 2.5	ND	ND

Linkage analysis was used to assess the types of polysaccharide linkages present in the mucilage samples (Table 2). This revealed low amounts of pectins (homogalacturonan, rhamnogalacturonan I/II; Supplementary Table S4) consistent with the monosaccharide analysis. Linkage analysis also identified low levels of 1,4-Glc, possibly derived from a small amount of cellulose in several of the samples (see Supplementary Table S4 at *JXB* online). The higher frequency of 1,4-Glc linkages in *P. coronopus* and *P. major* was consistent with the intense calcofluor white patterns identified for the same species. An acetic/nitric acid assay indicated traces only in *P. coronopus* extruded seed mucilage (Supplementary Fig. S2).

**Table 2.** *Polysaccharide summary of methylation linkage analysis on hot water-extracted* Plantago *mucilage (mol%*)

	*P. ovata*	*P. coronopus*	*P. cunninghamii*	*P. debilis*	*P. lanceolata*	*P. major*	*P. varia*
Arabinan	1.6	14.4	5.6	3.9	2.4	1.0	4.6
Type I AG	0.1	0.3	0.0	0.0	0.5	0.0	0.0
Type II AG	0.8	2.9	0.0	0.0	4.7	0.6	1.0
Homogalacturonan	0.2	0.4	0.2	0.8	1.0	0.3	1.2
RG I/II	0.0	0.6	0.2	0.3	0.1	0.0	0.0
3,4-Glucan	0.0	0.0	0.0	0.0	0.0	0.0	0.0
Heteroxylan	95.9	45.6	92.6	64.7	88.1	88.5	89.3
Heteromannan	0.0	1.2	0.0	0.2	0.2	0.4	0.1
Xyloglucan	0.8	4.4	0.0	0.4	0.3	1.0	0.3
Cellulose	0.5	12.9	0.1	1.7	1.3	6.9	1.1
Callose	0.0	5.8	0.1	22.0	0.4	0.8	1.7
Unassigned	0.1	11.4	1.2	6.0	1.0	0.4	0.5

Methylation linkage analysis identified heteroxylan in all seven *Plantago* species (Table 2). *P. ovata* data were consistent with previously published findings (Fischer *et al.*, 2004; Saghir *et al.*, 2008), indicating a heavily branched heteroxylan to be the main mucilage component, constituting approximately 95.5 mol% of total polysaccharide mass. The frequency of heteroxylan linkages varied between the remaining six species, with *P. cunninghamii* (92.6 mol%) containing a similar abundance to *P. ovata*, and *P. coronopus* containing the least amount (only 45.6 mol%). The type and frequency of heteroxylan linkages also varied considerably between species. 1,4-Xyl*p*, likely derived from an unsubstituted xylan backbone, comprised over 17% of the linkages in *P. cunninghamii* and over 11% in *P. lanceolata*. By contrast, only 0.8% and 1.6% of linkages in *P. coronopus* and *P. ovata*, respectively, corresponded to the same 1,4-Xyl*p* linkage. The degree of backbone substitution (i.e. 1,2,4-Xyl*p*, 1,3,4-Xyl*p* and 1,2,3,4-Xyl*p*) also varied. Similar to *P. debilis* and *P. varia*, over 18% of linkages in *P. ovata* were 1,2,4-Xyl*p*. By contrast, only 6.1% and 8.5% of the 1,2,4-Xyl*p* linkages were present in *P. lanceolata* and *P. cunninghamii*, respectively. A principal component analysis (PCA) plot based on the frequency of different xylan-associated linkages suggested that heteroxylan structure appears to vary somewhat independently of phylogenetic species relationships (see Supplementary Fig. S3 at *JXB* online).

### Xylan structure varies in the extruded seed mucilage of *Plantago* species

**Fig. 2.**
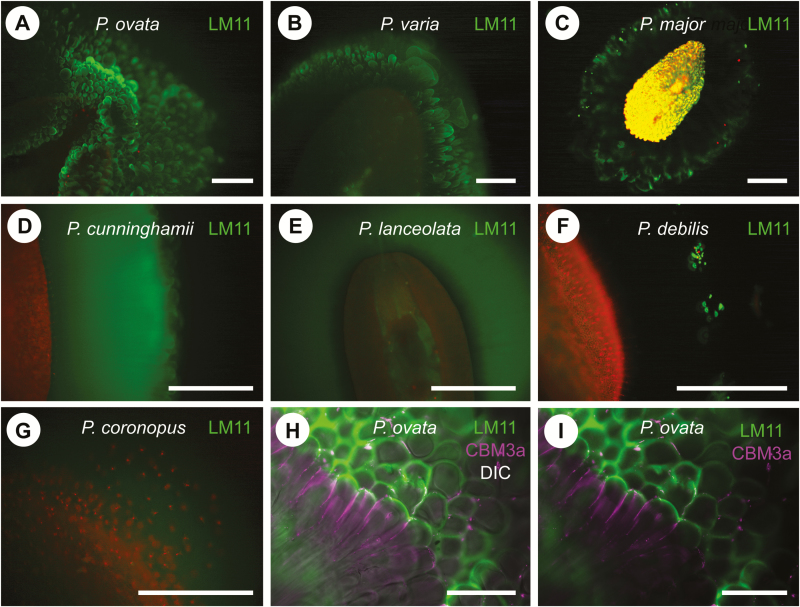
Whole-mount immunostaining of *Plantago* seeds identifies differences in LM11-antigenicity. (A–G) whole-mount seeds immunostained with LM11 antibody, raised against wheat arabinoxylan. LM11 labelling is shown in green, while red is indicative of propidium iodide staining. (A) Imbibed *P. ovata* seed showing characteristic anemone-like labelling at the mucilage periphery. (B) *P. varia*. (C) *P. major.* (D) *P. cunninghamii* showing an even distribution of LM11-labelling throughout the extruded seed coat mucilage. (E) *P. lanceolata.* (F) *P. debilis* showing only punctate spots of LM11-labelling in the mucilage. (G) *P. coronopus* was not labelled by LM11 antibody. (H, I) Magnified view of mucilage morphology in *P. ovata*, showing an overlay between LM11 labelling (green), CBM3a labelling (magenta) and differential interference microscopy (DIC). Cellulose is not prevalent in *P. ovata* mucilage, yet CBM3a recognizes an unknown epitope proximal to the seed coat relative to LM11 labelling. Bar A–G: 500 µm; H, I: 60 µm.

To assess the distribution of xylan in the seed mucilage of the different *Plantago* species a monoclonal antibody raised against wheat arabinoxylan, LM11 (McCartney *et al.*, 2005), was tested in whole-mount immunolabelling assays. Variable labelling patterns were detected (Fig. 2): anemone-like (*P. ovata*, *P. varia*, *P. major*; Fig. 2A–C), homogeneous (*P. cunninghamii*, *P. lanceolata*; Fig. 2D, E), speckled (*P. debilis*; Fig. 2F) and no labelling (*P. coronopus*; Fig. 2G). A CBM3a probe that recognizes several polysaccharides including cellulose and xyloglucan was used in a double labelling experiment with LM11 in *P. ovata*. This confirmed that antibodies are able to penetrate the mucilage since the different polysaccharides bound by CBM3a, compared with LM11, were arranged closer to the seed coat in a perpendicular orientation relative to its surface (Fig. 2H, I).

**Fig. 3.**
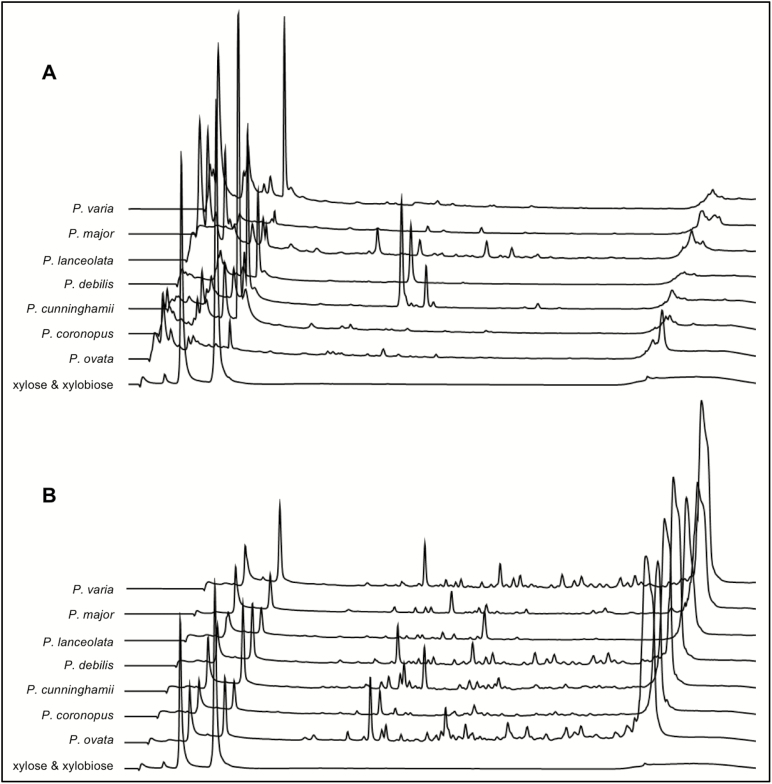
Xylanase-dependent oligosaccharide profiles reveal differences in the digestibility of seed coat mucilage from different *Plantago* species. Extruded seed mucilage was subjected to GH10 xylanase digestion. (A) untreated mucilage. (B) mucilage samples pre-treated with trifluoroacetic acid (TFA). Only *P. cunninghamii*, *P. debilis*, and *P. lanceolata* showed cleaved products (oligosaccharides) when incubated with the GH10 xylanase without TFA pre-treatment. All mucilage samples produced different oligosaccharide maps when pre-treated with TFA. The chromatograms have been off-set to aid interpretation. Elution times for xylose and xylobiose 2.5 min and 3.25 min, respectively.

Enzyme hydrolysis and oligosaccharide mapping investigated whether differences in heteroxylan structure, predicted by linkage and immunolabelling analyses, might confer differences in digestibility. Digestion of mucilage samples with a GH10 endoxylanase released oligosaccharides from *P. cunninghamii*, *P. lanceolata*, and *P. debilis* (Fig. 3A), but not from *P. ovata*, *P. coronopus*, *P. major*, or *P. varia*. Mild TFA was used as a pre-treatment to remove steric hindrance potentially caused by backbone substitutions (Fig. 3B). When pre-treated with TFA, all samples produced distinctive oligosaccharide profiles, including those that were previously resistant to digestion (Fig. 3B). Taken together, the compositional and structural data indicate that in general a highly substituted form of heteroxylan is present in these *Plantago* species but some, notably *P. cunninghamii*, *P. lanceolata*, and *P. debilis*, appear to contain significantly less substitutions overall. The differential substitution levels between species therefore provide a resource to identify molecular mechanisms by which *Plantago* heteroxylan structure is defined.

### Gene families involved in polysaccharide biosynthesis are transcribed in *P. ovata* and *P. cunninghamii* seed coat tissues

**Fig. 4.**
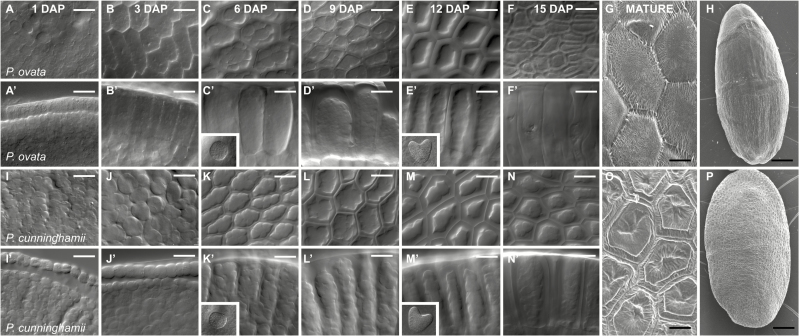
Seed coat development in *P. ovata* and *P. cunninghamii* follows a similar time course after pollination. (A–F′, I–N′) Developing seeds were collected and cleared whole-mount in Hoyer’s solution. (G, H, O, P) Scanning electron micrographs of mature seeds. (A–F) Top-down views of the *P. ovata* seed coat at 1, 3, 6, 9, 12 and 15 d after pollination (DAP). Starch accumulation is evident at 6 DAP while the characteristic shape of the seed coat epidermis becomes apparent at 12 DAP. (A′–F′) Side-on views of the *P. ovata* seed coat at 1, 3, 6, 9, 12 and 15 DAP. Globular-stage embryos are identified at 6 DAP while heart-shaped embryos are detected at 12 DAP. The epidermal cells rapidly elongate during early stages before filling with starch. Breakdown of starch is apparent from 9 to 15 DAP. (G, H) Two different types of morphology are detected in seed coat epidermal cells in mature *P. ovata* seeds, possibly dependent upon contact with the surrounding carpel tissues during maturation. (I–N) Top-down views of the *P. cunninghamii* seed coat at 1, 3, 6, 9, 12 and 15 DAP. Starch accumulation is evident at 6 DAP while the characteristic shape of the seed coat epidermis becomes apparent at 9–12 DAP. (A′–F′) Side-on views of the *P. cunninghamii* seed coat at 1, 3, 6, 9, 12 and 15 DAP. Similar to *P. ovata*, globular-stage embryos are identified at 6 DAP while heart-shaped embryos are detected at 12 DAP. The epidermal cells rapidly elongate, although not as fast as *P. ovata*, during early stages before filling with starch. Breakdown of starch is apparent from 9 to 15 DAP. (G, H) Only one type of morphology is detected in seed coat epidermal cells in mature *P. cunninghamii* seeds. Bar: A–N′: 20 µm, G and O: 20 µm, H and P: 500 µm.

Two of the characterized diploid *Plantago* species, *P. ovata* and *P. cunninghamii*, contained a similar abundance of heteroxylan-derived linkages in the extracted mucilage (96.9% and 92.6%, respectively) but showed distinct differences in individual linkage frequency and backbone substitution. To assess the molecular basis for these differences, we first examined seed coat development from 1 to 16 DAP in both species to determine if developmental timing was similar (Fig. 4). In both species, epidermal seed coat cells (Fig. 4A, I) began expanding after pollination (Fig. 4B, J) to adopt a characteristic elongated shape by 6 DAP (Fig. 4C, K). The cells accumulated amorphous material, most likely to be starch (Fig. 4D, L) and by 12 DAP (Fig. 4E, M) began to assume a morphology similar to the mature seed coat (Fig. 4F–H, N–P). Embryo development was similar in both species, with late globular- and heart-stage embryos detected at 6 and 12 DAP respectively (Fig. 4C′, E′, K′, M′), and mature linear torpedo-shaped embryos at 16 DAP. These data suggest that seed coat tissues develop similarly in the two species.

To confirm when heteroxylan synthesis occurs during this time course, developing *P. ovata* seeds were sectioned and analysed by toluidine blue staining and LM11 immunolabelling (see Supplementary Fig. S4 at *JXB* online). The structure of seed coat cells was remarkably similar to that described by Hyde (1970). At 7 DAP, seed coat epidermal cells had elongated and contained abundant starch granules (Supplementary Fig. S4A–D), while transmission electron microscopy indicated a limited amount of internal LM11 labelling (Supplementary Fig. S4E–G). By 12 DAP, seed coat epidermal cells had often ruptured during chemical fixation, releasing material that stained heavily with toluidine blue (Supplementary Fig. S4H–J). Intact epidermal cells at this stage showed abundant internal labelling with LM11 (Supplementary Fig. S4K), consistent with the accumulation of a heteroxylan-rich mucilage. Labelling was absent from the collapsing protoplast and sub-epidermal cells (Supplementary Fig. S4K, L). This suggests that the 1–16 DAP time course can be used to further investigate molecular details of mucilage heteroxylan biosynthesis in the anatomically similar seed coats of *P. ovata* and *P. cunninghamii*.

A general transcriptome was assembled for *P. ovata* using the Illumina HiSeq platform. RNAseq data were generated from pooled tissue samples including developing cotyledons, roots, inflorescences, flowers, leaves, stems, ovules, integuments and seeds, and assembled into a contig set using CLC Genomics Workbench v8.0. This transcriptome comprised 49 329 contigs with an N50 of 1314 bp. To identify molecular differences between *P. ovata* and *P. cunninghamii* that might explain differences in their mucilage heteroxylan structure, integument/seed coat tissue was harvested for both species at 12–14 DAP and RNA was sequenced. This tissue was found to be enriched with xylan-associated biosynthetic genes (see Supplementary Fig. S5B at *JXB* online). RNAseq reads from *P. ovata* were mapped to the *P. ovata* contig set to quantify gene espression of seed coat-enriched sequences, while the *P. cunninghamii* reads were assembled and mapped *de novo* to create a seed coat transcriptome that consisted of 30 419 contigs; N50=1084 bp. Contigs from both species were annotated through BLAST searches against Genbank and Arabidopsis databases. In total, ~45% of the *P. ovata* contigs and ~58% of the *P. cunninghamii* contigs could be annotated by comparison to Arabidopsis based on an e-value cutoff of <10^–3^.

Contig sets were filtered to identify specific gene families implicated in polysaccharide biosynthesis, and BLAST searches and alignments were used to capture all homologous family members. The GT2 CELLULOSE SYNTHASE (CESA) enzymes are involved in cellulose biosynthesis during most stages of growth and development, and therefore act as a reference for general cell wall related activity. A total of eight *CesA* contigs were identified in the *P. ovata* contig set (Supplementary Figs S5A and S6A), and five of these were highly abundant in the developing seed coat tissues, including the homologues of Arabidopsis *CesA1*, *3*, *4*, *6* and *8* (Supplementary Figs S5B and S6A). In *P. cunninghamii*, seven *CesA* sequences were identified in the seed coat contig set, and homologues of Arabidopsis *CesA1*, *3*, *6*, *8* and *9* were most abundant (Supplementary Figs S5A and S6A). *P. cunninghamii* sequences grouped closely with the *CesA* sequences from *P. ovata* in a phylogenetic tree, and in the majority of cases matched a single Arabidopsis *CesA* sequence (see Supplementary Fig. S5A at *JXB* online). No homologue of *PoCesA7* was identified in the *P. cunninghamii* transcriptome indicating it may be abundant in tissues other than the seed coat. Apart from some differences in transcript abundance for the *CesA4* and *CesA9* homologues (Supplementary Fig. S6A), the number of *CesA* genes and their transcript accumulation profiles appeared to be very similar in the two *Plantago* species.

Genes showing homology to *IREGULAR XYLEM 10* (*IRX10*) from Arabidopsis, a member of the GT47 family, have been implicated in synthesis of the xylan backbone (Jensen *et al.*, 2014; Urbanowicz *et al.*, 2014). *IRX10* homologues are highly abundant in the developing seed coat of *P. ovata*, where six unique *IRX10-L* genes were previously identified as being expressed (Jensen *et al.*, 2013). Analysis of the core *P. ovata* contig set in this study identified at least 42 contigs from the GT47 family, of which six are closely related to *IRX10*, and four were abundant in the seed coat sample (*PoIRX10_2*, *3*, *4* and *7*; Supplementary Figs S5B, C and S6A). *PoIRX10_1* (Jensen *et al.*, 2013) was not detected in any *P. ovata* tissue sample analysed in this study, while *PoIRX10_6* was most abundant in bract tissues (see Supplementary Fig. S5B at *JXB* online). In *P. cunninghamii* a total of 27 contigs from the GT47 family were identified in the developing seed coat. Of these, four were closely related to *IRX10*, and based on phylogenetic analysis were annotated as *PcIRX10_2*, *2L*, *3* and *5* (Supplementary Fig. S5C)*. PcIRX10_2*, *2L* and *3* were most abundant in the seed coat (Supplementary Fig. S6A). Therefore, at least three *IRX10* genes are abundant in the seed coat tissues of *P. ovata* and *P. cunninghamii*, and the *IRX10_2* and *3* orthologues show the closest interspecific conservation with regards to transcription.

### Identification of GT61-like sequences in *P. ovata* and *P. cunninghamii*

**Fig. 5.**
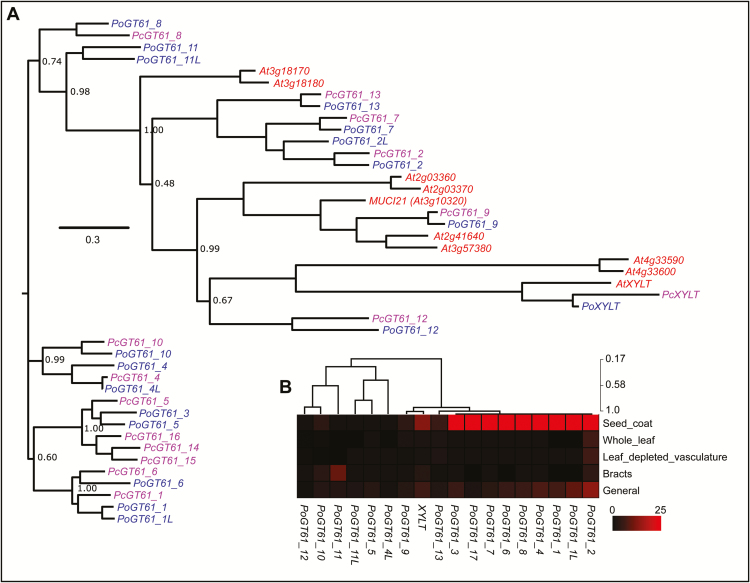
Analysis of the *Plantago* glycosyltransferase (GT61) family. (A) Coding sequence relationships between GT61 sequences from *Plantago ovata*, *Plantago cunninghamii* and *Arabidopsis thaliana*. (B) Heat map showing the relative transcript abundance (RPKM) of GT61 sequences in different *P. ovata* tissues including seed coat (12–14 DAP), whole leaf, whole leaf depleted of vascular tissues, bracts and a general tissue pool. Samples were ordered using the hierarchical clustering function in MeV with default parameters but no sample ordering. The maximum cut-off was drastically reduced to 25 in order to visualize expression in tissues other than the seed coat.

GT61 family members have been implicated in the substitution of xylan polysaccharides in species such as rice and wheat (Anders *et al.*, 2012; Chiniquy *et al.*, 2012), and are therefore attractive candidates driving structural differences in heteroxylan identified between *P. ovata* and *P. cunninghamii*. A total of 18 GT61 contigs were identified in the *P. ovata* transcriptome of which nine were predominantly detected in the developing seed coat (Fig. 5 and Supplementary Fig. S6A). In comparison, 15 GT61 sequences were identified in the *P. cunninghamii* dataset of which 10 were abundant in seed coat tissues (Fig. 5 and Supplementary Fig. S6A). The GT61 family is therefore considerably expanded in two members of the Plantaginaceae compared with other eudicots such as Arabidopsis. Phylogenetic analysis of GT61 sequences from *P. ovata* and *P. cunninghamii* categorized them into multiple clades (Fig. 5A). In many cases a 1:1 relationship was identified for GT61 genes from the two *Plantago* species, similar to that observed for *CesA* and *IRX10* families (Supplementary Fig. S5A, C. However, multiple clades were identified where an additional highly similar GT61 sequence was present in *P. ovata* compared with a single *P. cunninghamii* orthologue. This included *PoGT61_1*, *PoGT61_4*, *PoGT61_3/5* and *PoGT61_7/17*. Conversely, the *PcGT61_14*, *15* and *16* sequences lacked direct orthologues in *P. ovata*. Therefore, whilst similar numbers of GT61 sequences are found in the *P. ovata* and *P. cunninghamii* seed coat samples, duplications of specific GT61 sequences are found in both species and are particularly evident in the case of *P. ovata*.

### GT61 genes show dynamic transcriptional changes during seed coat development in *P. ovata* and *P. cunninghamii*

We considered dynamic changes in transcript profiles, and hence enzyme abundance of different GT61 sequences, might impact heteroxylan structure in the two species. A developmental series, from 1 to 16 DAP, of integument tissue was harvested (Fig. 4) and used to generate gene expression profiles by qPCR. To assess quality of the tissue series, profiles were generated for a set of four orthologous *CesA* genes from both species (see Supplementary Fig. S6B at *JXB* online). *PoCesA1*, *PoCesA3* and *PoCesA6* were barely transcribed during the first 12 days of seed coat development but then peaked dramatically at 13–14 DAP. *PoCesA9* was expressed at very low levels across the same developmental time course. For *P. cunninghamii PcCesA1*, *PcCesA3* and *PcCesA9* transcripts gradually increased from 8 DAP peaking at 12 DAP, while *PcCesA6* was barely expressed above background (Supplementary Fig. S6B). The late transcript peak suggests that polysaccharide biosynthesis is following a similar temporal pattern in both species, and that an important phase of biosynthesis occurs relatively late during *Plantago* seed coat development. The *Plantago IRX10_2*, *IRX10_3* and *IRX10_4* genes were also analysed. *PoIRX10_2* and *PoIRX10_3* showed prominent transcript peaks at 13–14 DAP in *P. ovata*, while *PcIRX10_3* was high at 12–13 DAP in *P. cunninghamii* (Supplementary Fig. S6C). The late peaks of *CesA* and *IRX10* transcript coincide with the accumulation of heteroxylan in the *P. ovata* seed coat epidermal cells (Supplementary Fig. S4), and suggest these are likely to be the key stages of development for mucilage accumulation; *CesA* genes are possibly involved in the development of mucilage storage cell structure and *IRX10* genes influence xylan synthesis in both species (Supplementary Fig. S6C).

**Fig. 6.**
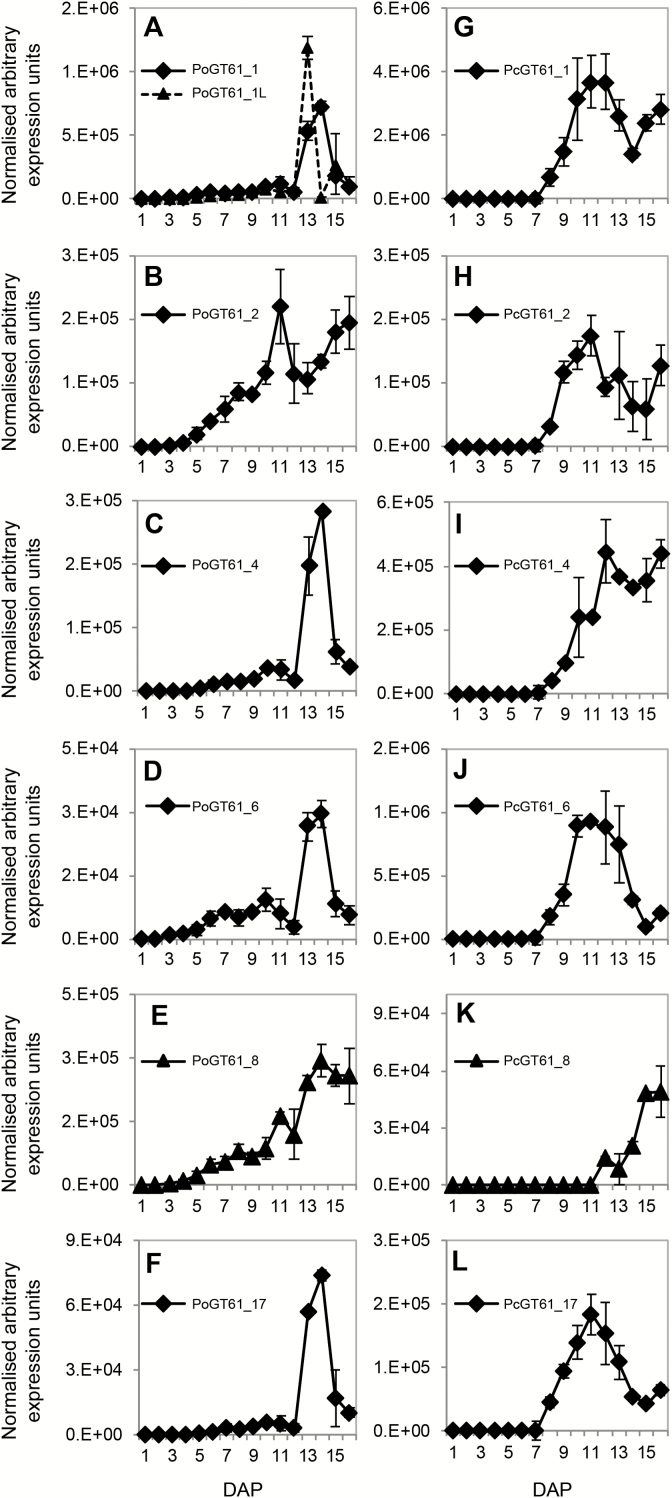
Transcriptional profiles of selected homologous *Plantago* GT61 family sequences during seed coat development in *P. ovata* (A–F) and *P. cunninghamii* (G–L). In each plot the *y*-axis shows expression values that were normalized within each species. Error bars show standard deviation.

**Fig. 7.**
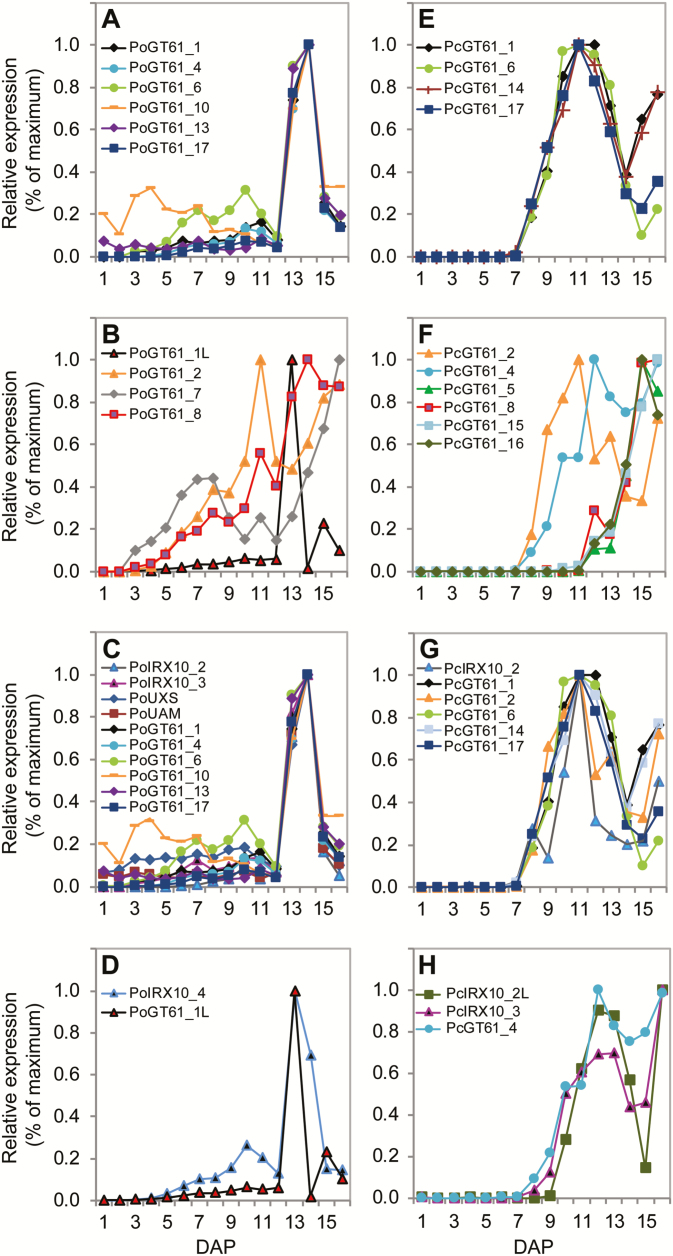
Percentage plots of *Plantago* GT61 family sequences during seed coat development in *P. ovata* (A–D) and *P. cunninghamii* (E–H). In each plot the *y*-axis shows percentage expression relative to the maximum for each sequence. Error bars show standard deviation.

Transcript profiles were generated for selected GT61 sequences across seed coat development for both *Plantago* species (Figs 6 and 7 and Supplementary Fig. S7). Consistent with RNAseq results, *PoGT61_1* and *1L* were the most abundant GT61 transcripts in *P. ovata* (Fig. 6A) and similar to *PoGT61_4*, *6*, *7*, *10* and *16*, showed distinct peaks in transcript abundance at 13–14 DAP (Figs 6 and 7 and Supplementary Fig. S7). *PoGT61_2* accumulated earlier during seed coat development and peaked at 11 DAP (Fig. 6B), somewhat similar to *PoGT61_8* (Fig. 6E), which started accumulating at 4 DAP before peaking at 14 DAP. *PoGT61_7* was distinct, showing a peak in transcription at 3–10 DAP before decreasing and thereafter increasing to 16 DAP (see Supplementary Fig. S7 at *JXB* online). When values were normalized relative to the maximum expression for each gene, five different transcript patterns were identified for the GT61 sequences in *P. ovata* (Fig. 7A, C, E, G). The majority of *PoGT61* genes showed remarkably similar profiles to the *PoIRX10_2*, *PoIRX10_3*, *UDP-xylose synthase* (*PoUXS*) and *UDP-arabinose mutase* (*PoUAM*) genes (Fig. 7E) that are implicated in xylan biosynthesis (Rennie and Scheller, 2014).

Although the patterns tended to spread over a broader time period, the *PcGT61_1*, *2*, *6*, *8* and *17* sequences showed similar transcript profiles relative to their closest *P. ovata* homologues (Fig. 6G–L). *PcGT61_1* was the most abundant *GT61* in *P. cunninghamii* (Fig. 6G). At least five different transcript patterns were observed for the *PcGT61* gene family (Figs 6G–L and 7E–H and Supplementary Fig. S 7). Two patterns were prominent: four genes peaked at ~11 DAP (*PcGT61_1*, *6*, *14* and *17*; Fig. 7F) and four at ~15 DAP (*PcGT61_5*, *8*, *15* and *16*; Fig. 7D). The five *PcGT61* genes that peaked at 11 DAP showed the most similar profile to the putative xylan synthase *PcIRX10_2* (Fig. 7F), suggesting they may play a role in xylan substitution during biosynthesis.

In summary, these data indicate that during seed coat development, orthologous *GT61* genes in *P. ovata* and *P. cunninghamii* show similar expression dynamics in the majority of cases. Many of the *GT61* transcript profiles show a close association with the putative xylan biosynthetic machinery, and this is particularly evident in the case of *P. ovata*. *GT61_1* is the most abundant GT61 in both species, but is represented by two highly abundant sequences in *P. ovata*. At least five of the 12 *P. ovata* GT61 sequences appear to have a closely related or duplicated sequence, which contrasts to the *CesA* and *IRX10* gene families, as well as the GT61 family in *P. cunninghamii*.

## Discussion

Natural genetic variation is a powerful resource for identifying genes and pathways involved in regulation of plant growth and development. Variation in primary cell wall composition (Mouille *et al.*, 2006) and mucilage polysaccharide release (North *et al.*, 2014), have been used to identify QTLs and underlying genes in Arabidopsis that influence polysaccharide synthesis and structure. Increased availability of genomic methods makes exploiting genetic variation more useful for traditionally non-model species, permitting the characterization and identification of molecular cues underlying trait variation.

In this study a variety of techniques were used to characterize the seed mucilage of seven related *Plantago* species. Previous studies in *Plantago* have focused on the seed mucilage of *P. ovata*, an important therapeutic tissue composed predominantly of a heteroxylan polysaccharide rich in xylose and arabinose (Edwards *et al.*, 2003; Fischer *et al.*, 2004; Guo *et al.*, 2008; Saghir *et al.*, 2008). Similar to *P. ovata*, xylose and arabinose were prominent in the extracted seed mucilage of the other six *Plantago* species analysed (Table 1). Linkage analysis suggested that the majority of these sugars are derived from heteroxylan polysaccharides, but the abundance of each sugar and type of heteroxylan linkage varied between species (Table 2). Differences in composition were generally reflected in the variable LM11 antibody labelling (Fig. 2). Although LM11 was raised against wheat arabinoxylan (McCartney *et al.*, 2005), mucilage from six of the seven *Plantago* species showed antigenicity, albeit with four different staining patterns and varying levels of avidity, indicating that some heteroxylan substitutions in *Plantago* are sufficiently similar to the arabinose substitutions in wheat arabinoxylan. Using ruthenium red, at least five different staining patterns were observed for the *Plantago* species (Fig. 1). Although no other species displayed the complex cloud-like mucilage observed for *P. ovata*, a common feature was a transparent, LM11-negative layer that separated the seed coat from an intensely stained peripheral region of acidic or LM11-positive polysaccharides. The transparent layer did not stain with CBM3a and linkage analysis identified only low levels of 1,4-glucose, suggesting that unlike Arabidopsis mucilage (Sullivan *et al.*, 2011), little cellulose is present. Guo *et al.* (2008) used different extraction methods to address the composition of *P. ovata* mucilage layers, finding subtle differences in monosaccharide composition. Consistent with our data using different extraction methods (J. Phan, L. Yu, unpublished data), it appears likely that the different layers in *P. ovata* are composed of the same polysaccharides but with variable structures and rheological properties.

The complex nature of *Plantago* mucilage heteroxylan was evident from the methylation linkage data (Table 2 and Supplementary Table S3) and digestibility assays (Fig. 3). GH10 xylanases hydrolyse β-(1,4)-linkages next to single or double substituted xylose units, as long as two unsubstituted xylose residues are found between the substituted residues (Pollet *et al.*, 2010). Addition of a GH10 xylanase liberated oligosaccharides from untreated mucilage samples in three species, and for *P. cunninghamii* and *P. lanceolata* these data are consistent with these mucilages containing the highest proportion of unsubstituted xylan backbone as determined by linkage analysis. PCA multivariate plots suggested the degree of xylan substitution was a major difference between the species, and was independent of phylogenetic relationships between species as determined by ITS sequence comparisons (see Supplementary Fig. S3 at *JXB* online). While *P. varia*, *P. debilis*, and *P. cunninghamii* were closely related based on phylogeny, their mucilage composition, LM11 immunogenecity and heteroxylan structures were distinct, and so too were *P. ovata* and *P. lanceolata*. Although *P. ovata* and *P. cunninghamii* were chosen for further analysis based on their ploidy (both diploid), similar mol% of heteroxylan, equivalent seed coat development but clear differences in heteroxylan substitution, other species could be used to characterize specific genes contributing to mucilage composition and polysaccharide structure in future studies.

Consistent with the linkage data presented here (Table 2 and Supplementary Table S4), Fischer *et al.* (2004) reported that the β-(1,4)-Xyl*p* backbone of *P. ovata* is decorated with single *β*-(1,2)-xylosyl substitutions and sidechain structures including α-Ara*f*-(1,3)-β-Xyl*p*-(1,3)-Ara*f*. In comparison with *P. ovata*, *P. cunninghamii* mucilage contained fewer 1,2,4-Xyl*p* linkages (i.e. substitution at C2), slightly more 1,2,3,4-Xyl*p* linkages (i.e. C2 and C3 di-substitution), a similar abundance of 1,3-Xyl*p* linkages (i.e. components of substituent sidechains), and a similar number of terminal xylose linkages. *P. cunninghamii* also showed fewer 1,3-Ara*f* (i.e. components of substituent side chains) and terminal arabinose linkages. Therefore several enzyme activities are likely to differ between the two species. In general, the *P. ovata* backbone is more substituted than that of *P. cunninghamii*, with preference for a substitution at C2 with either Xyl*p* or Ara*f*. Both species show a similar preference for substitution at C3 but when *P. cunninghamii* has a Xyl*p* substitution at C2, the C3 position is more likely to be substituted compared with *P. ovata*. Taken together, this suggests that the enzyme activities in *P. ovata* must show (i) a greater preference for backbone substitution, (ii) a combination of xylosyltransferase and arabinosyltransferase activity, and (iii) a preference for substituent chain elongation rather than di-substitution. By contrast, the enzymes in *P. cunninghamii* create less frequent backbone substitutions, typically via xylosyltransferase activity, at the C2 and/or C3 positions.

Identification of several monocot GT61 enzymes as β-(1,2)-xylosyltransferases or α-(1,3)-arabinosyltransferases (Anders *et al.*, 2012; Chiniquy *et al.*, 2012) suggests that GT61 family genes may contribute to different xylan structures identified between *P. ovata* and *P. cunninghamii*. Monocot species generally contain more GT61 genes than eudicots, congruent with monocot xylan being heavily substituted with arabinosyl residues (Anders *et al.*, 2012), while eudicot species typically contain xylan substituted with glucuronic acid mediated by GT8 enzymes (Scheller and Ulvskov, 2010). Some eudicot GT61 sequences have been shown to function in protein glycosylation, including Arabidopsis XYLT (Strasser *et al.*, 2000). *P. ovata* is unusual among eudicots in containing a greater number of GT61 genes (Jensen *et al.*, 2013) and *P. cunninghamii* is similar (Fig. 5A), suggesting that expansion of the GT61 family may be a general feature of the *Plantago* genus. Jensen *et al.* (2013) previously identified seven GT61 sequences, *GT61_1* to *GT61_7*, in the integument tissue of *P. ovata*. Here, 18 GT61 sequences were identified in a general *P. ovata* tissue set (including a single putative XYLT sequence), of which 14 were expressed, nine preferentially in the developing seed coat (Fig. 5B). Furthermore, 15 GT61 sequences were identified in *P. cunninghamii*, of which 10 were abundant in the developing seed coat. This indicates that in general, GT61 genes are highly abundant in the seed coat tissues of both species. Detailed phylogenetic analysis (Fig. 5A) and determination of their transcript profiles during 16 stages of seed coat development identified key differences between the sequences and species that may contribute to differences in xylan structure.

The first obvious difference was the high frequency of duplicated sequences in *P. ovata* that were represented by a single sequence in *P. cunninghamii* (Fig. 5A). This included the pairs of *PoGT61_1* and *1L*, *PoGT61_4* and *4L*, *PoGT61_3* and *5*, *PoGT61_11* and *11L* and *PoGT61_2* and *7*. Two of these pairs (*PoGT61_1/1L* and *PoGT61_2/7*) were analysed by qPCR during seed coat development and each pair showed a distinct expression profile (Fig 7A, C), suggesting that they may have adopted specialized roles during seed coat development. The *GT61_1* clade, for example, contained the most abundant *GT61* sequence in both species and was represented by two sequences in *P. ovata*. *PoGT61_1* showed a similar expression profile to *PoIRX10_3* (Fig. 7E), while *PoGT61_1L* showed a similar expression profile to *PoIRX10_4* (Fig. 7G). The different transcript profiles of two abundant *PoIRX10* genes and their associated *PoGT61_1* sequences suggests that different complexes might be acting on different substrates or at different stages during xylan biosynthesis. If these highly abundant *GT61_1* sequences directly act on the β-(1,4)-Xyl*p* backbone during biosynthesis, the extra copy of *GT61_1* may be one reason for the increased frequency of backbone substitution in *P. ovata*. Several groups have been able to demonstrate the requirement of such a complex (Zeng *et al.*, 2010, 2016; Jiang *et al.*, 2016), despite the lack of evidence of GT61s being present. The temporal patterning of the *Plantago* putative xylan synthesis components, IRX10 and GT61, together with the well characterized CESAs, is at least suggestive of the synergistic action of multiple enzymes to produce the final heteroxylan structure. Whether or not these putative components concatenate to form a final xylan synthesis complex will require further investigation.

The results also reveal a difference in the number of GT61 sequences showing a close association with a predicted *IRX10* xylan synthase gene in *P. ovata* and *P. cunninghamii*. *IRX10* homologues showed prominent peaks in expression at 13–14 DAP in *P. ovata* (Fig. 7E, G), coinciding with the accumulation of heteroxylan in seed coat epidermal cells (see Supplementary Fig. S4 at *JXB* online), and at 10–13 DAP in *P. cunninghamii* (Fig. 7F, H). Expression profiling of ten *PoGT61* sequences revealed that six (*PoGT61_1*, *4*, *6*, *10*, *13* and *17*) showed a close association with the *PoIRX10_3*, *PoUXS* and *PoUAM* sequences (Fig. 7E). Only four of the ten analysed *PcGT61* sequences (*PcGT61_1*, *6*, *14* and *17*) showed a close association with *PcIRX10_2* in *P. cunninghamii* (Fig. 7F, G). Although the low abundance of several *P. ovata* sequences (e.g. *PoGT61_10* and *13*) may preclude a significant role in xylan synthesis, profiling data imply that more GT61s are expressed in concert with the xylan biosynthetic machinery in *P. ovata*. If co-expression of GT61s with *IRX10* is a key requirement for xylan substitution, this may explain structural differences between *P. ovata* and *P. cunninghamii*. Alternatively, but not mutually exclusively, xylan substitution may occur at different stages of xylan biosynthesis or seed coat development. Additional GT61 sequences showed transcriptional profiles that were distinct from *IRX10_3* or *IRX10_4*, but were highly conserved between *P. ovata* and *P. cunninghamii*. If these sequences are also involved in xylan substitution, they may be acting on different substrates independently of IRX10 activity. Further transcriptional and functional analysis of these sequences in parallel with the putative IRX10 homologues may reveal more details of their role in seed coat development and xylan biosynthesis.

Finally, GT61s that showed a similar transcription profile to *IRX10* genes differed slightly between the two species. *PoGT61_1*, *6* and *17* showed similar patterns in both species, indicating that the encoded proteins may fulfil similar functions (Fig. 7A, B). In contrast, *PcGT61_14* was located in a small subclade unique to *P. cunninghamii* (Fig. 5A) and was co-expressed with the dominant *PcGT61_1*, *6* and *17* sequences (Fig. 7B). The enzyme encoded by *PcGT61_14* may therefore be responsible for specific substitutions prevalent in *P. cunninghamii* heteroxylan but less so in *P. ovata*. In addition, *PoGT61_4* and *PcGT61_4* were both highly abundant in the seed coat (Fig. 6C, I) but displayed distinct patterns, such that *PoGT61_4* was co-expressed with *IRX10_3* (Fig. 7E) while *PcGT61_4* was not. *PoGT61_4* may therefore be a strong candidate to investigate in relation to the higher degree of backbone substitution in *P. ovata*.

In summary, seed mucilage from the seven *Plantago* species is rich in heteroxylan, but shows distinct differences in immunolabelling efficiency, xylanase digestibility and xylan-associated linkage abundance. Two species, *P. ovata* and *P. cunninghamii*, produce seed mucilage that contains a similar amount of heteroxylan but varying degrees of backbone substitution. Transcriptional analysis of integument tissues indicates that GT61 family sequences, implicated in xylan substitution, are highly abundant in both species but gene specialization and duplications are likely to be contributing to differences in heteroxylan structure. Furthermore, co-expression of GT61 sequences with putative IRX10 homologues indicates that xylan substitution may be occurring in concert with xylan synthesis, and that the occurrence of co-expressed GT61 genes may impact eventual mucilage xylan structure. Whether these diverse genes are acting as xylosyltransferases and/or arabinosyltransferases remains to be determined using systems for functional analysis such as floral dip transformation in *P. major* (Pommerrenig *et al.*, 2006) and *P. ovata* callus cultures (Gupta *et al.*, 2015; Talukder *et al.*, 2016). Details of GT61 transcripts and other putative components of the xylan synthase machinery will also prove to be a valuable resource when examining transcript data for the other species reported here, in addition to reverse-genetic screening mutant *P. ovata* populations for candidate genes implicated in mucilage composition and release.

## Supplementary data

Supplementary data are available at *JXB* online

Fig. S1. Ruthenium red staining of seed mucilage pre and post hot water extraction.

Fig. S2. Crystalline cellulose content in seeds and extracted seed mucilage of seven *Plantago* species.

Fig. S3. Comparison of *Plantago* species based on heteroxylan-associated linkages and internal transcribed spacer regions.

Fig. S4. Histological details of seed coat development and heteroxylan accumulation in *P. ovata*.

Fig. S5. Analysis of the *CesA* and *IRX10-like* genes in P. o*vata*, *P. cunninghamii* and Arabidopsis.

Fig. S6. RNAseq and qPCR analysis of selected *CesA*, *IRX10* and *GT61* sequences from *P. ovata* and *P. cunninghamii*.

Fig. S7. QPCR expression profiles of selected *P. ovata* and *P. cunninghamii* GT61 genes during seed coat development.

Table S1. Location from which *Plantago* seeds were obtained.

Table S2. Primers used for qPCR transcript analysis.

Table S3. Monosaccharide data showing mol% relative to the amount of mucilage per seed.

Table S4. Complete table of linkages present in the seed mucilage of seven *Plantago* species.

Supplementary Data

## Abbreviations:

CESA: CELLULOSE SYNTHASE;
DAP: days after pollination;
GT: glycosyltransferase;
IRX10: *IREGULAR XYLEM 10*;
RNAseq: RNA sequencing;
RP-HPLC: reverse phased high performance liquid chromatography;
TFA: trifluoroacetic acid.
