# Supplementary material for: Differences in glycosyltransferase family 61 accompany variation in seed coat mucilage composition in Plantago spp
Source: J Exp Bot. 2016 Nov 17;67(22):6481–95. doi: 10.1093/jxb/erw424 (PMC5181589; doi:10.1093/jxb/erw424)
Supplement: Supplementary Data [file supp_67_22_6481__index.html]

Differences in glycosyltransferase family 61 accompany variation in seed coat mucilage composition in Plantago spp. — Supplementary Data 

# Differences in glycosyltransferase family 61 accompany variation in seed coat mucilage composition in *Plantago* spp.

## Supplementary Data

Data files

- supplementary\_figures\_S1\_S7.pdf - Supplementary Data
- supplementary\_tables\_S1\_S4.xlsx - Supplementary Data
