# Supplementary material for: Differences in glycosyltransferase family 61 accompany variation in seed coat mucilage composition in Plantago spp
Source: J Exp Bot. 2016 Nov 17;67(22):6481–95. doi: 10.1093/jxb/erw424 (PMC5181589; doi:10.1093/jxb/erw424)
Supplement: Supplementary Data [file supp_erw424_supplementary_figures_S1_S7.pdf]

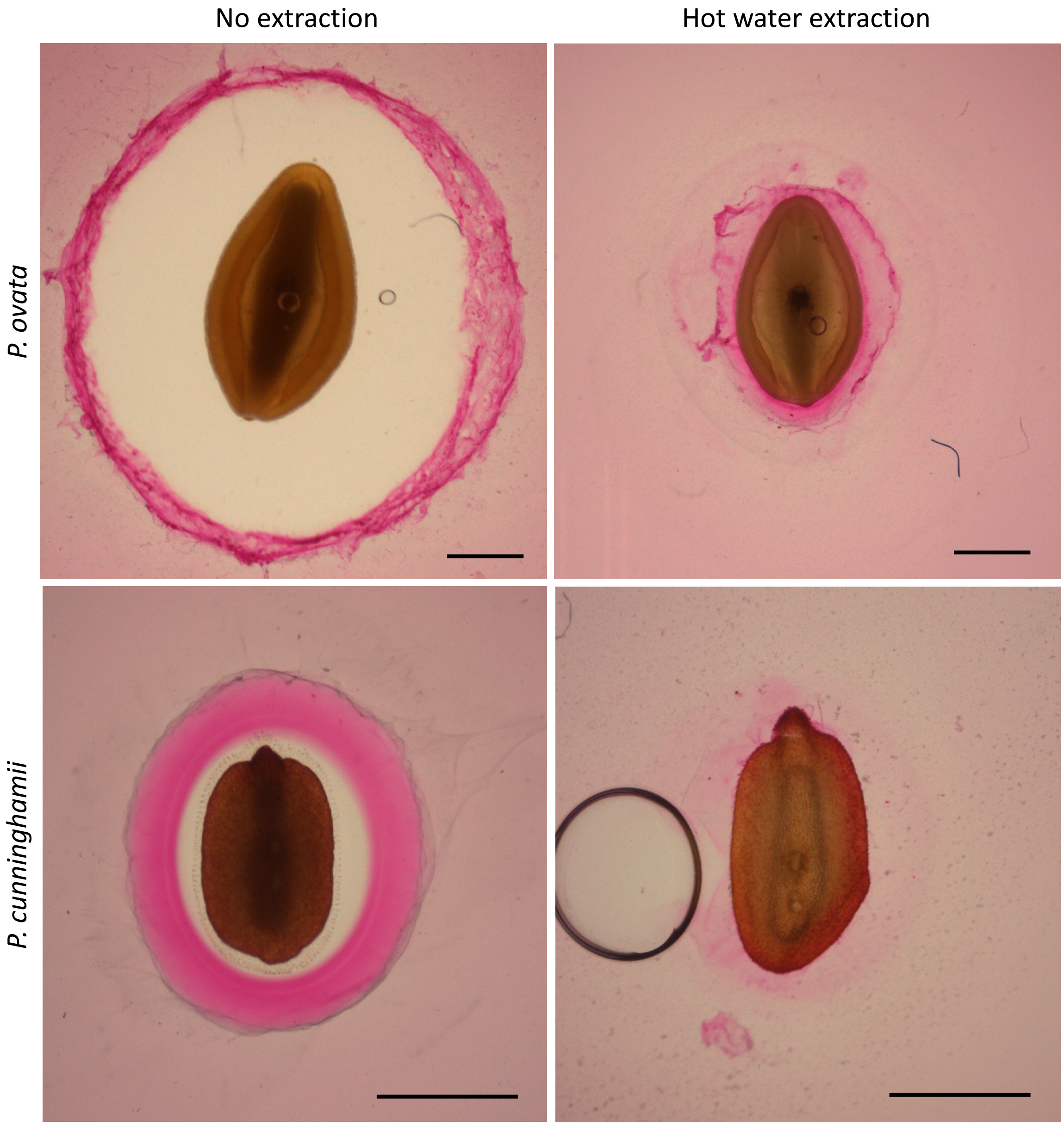

Fig. S1. *Plantago* seed mucilage stained with ruthenium red (0.01% w/v) with and without hot water extraction. For staining of seeds without mucilage extraction, seeds were first placed into water for 90 min and post stained for 30 min. Seed mucilage was extracted as stated in 'materials and methods' and stained as above. Hot water extraction of seed mucilage from *Plantago* species was able to liberate a majority of the mucilage from most species. *P. ovata* seed mucilage was also extracted using 0.2M KOH but monosaccharide analysis of the hot water extracted fraction and the KOH fraction showed insignificant compositional differences (J. Phan and L. Yu, unpublished). Bar = 1mm.

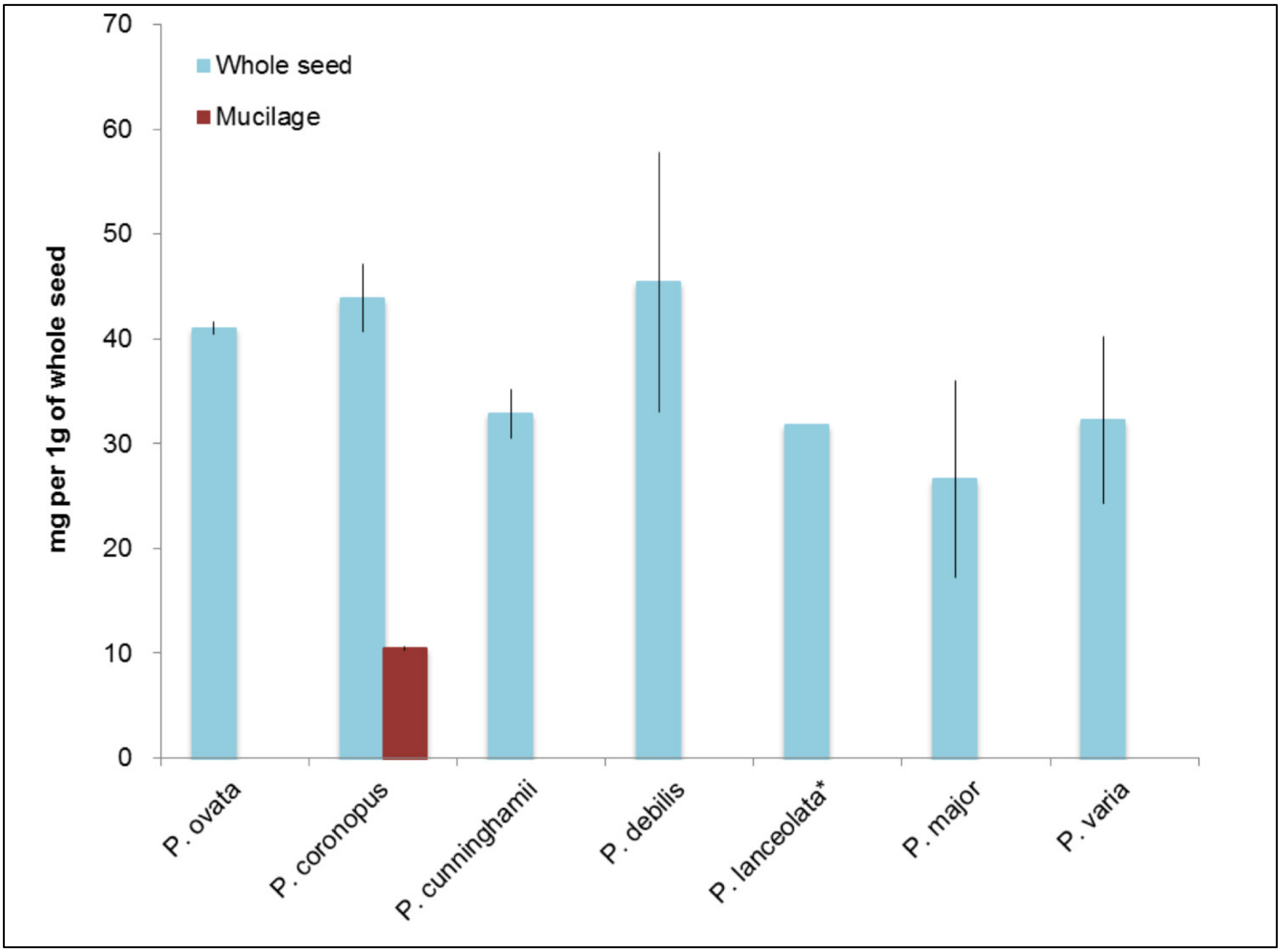

Fig. S2. Normalised crystalline cellulose levels obtained from 1 g of whole seed and mucilage extracted from 1 g of whole seed, determined as per Updegraff (1969). *Only one sample was available for crystalline cellulose determination. Error bars = SD (n = two).

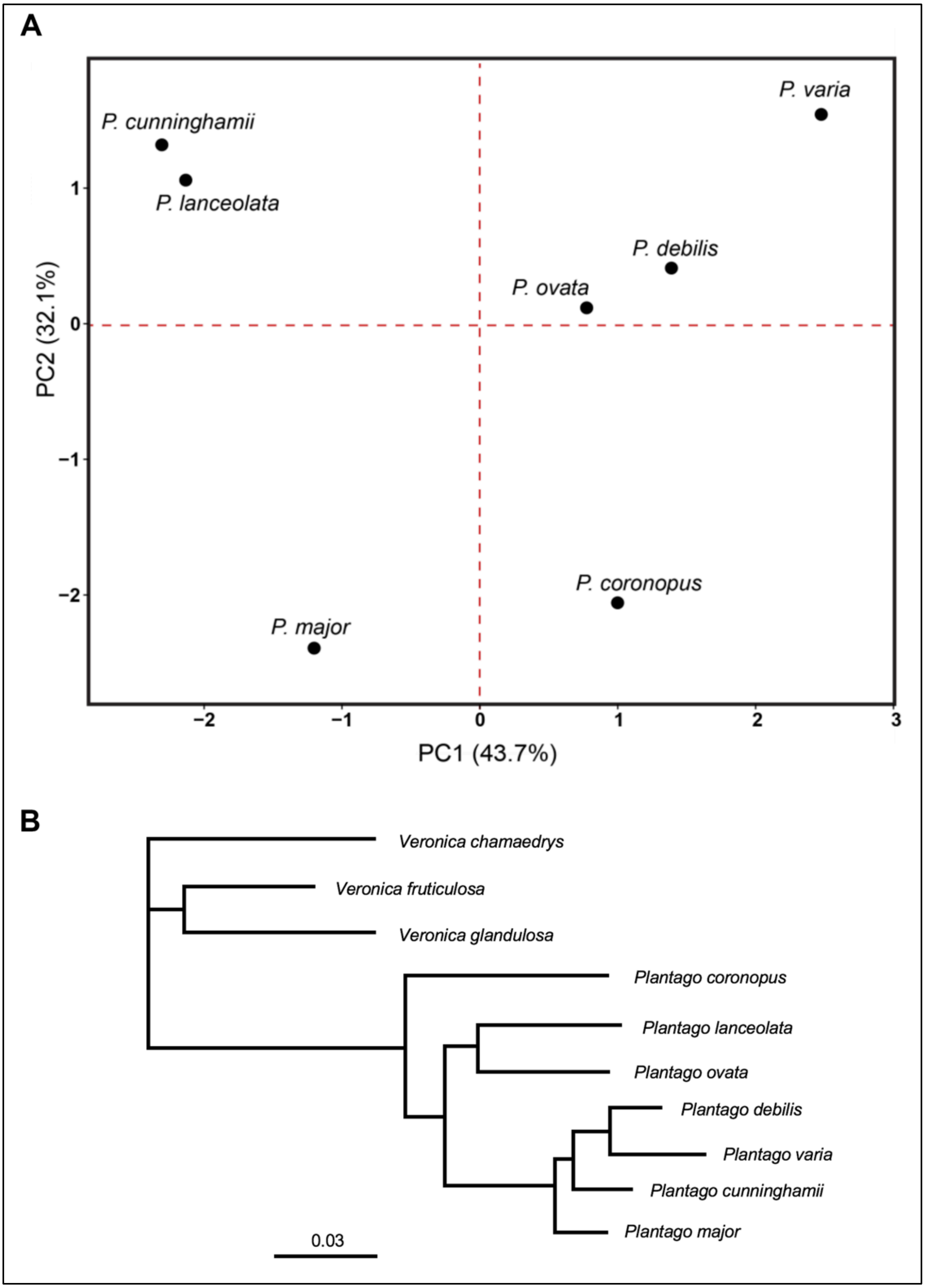

Fig. S3. Comparisons of *Plantago* species based on heteroxylan-associated linkages and internal transcribed spacer (ITS) regions. (**A**) PCA multivariate plot generated using the frequency of heteroxylan-associated linkages in *Plantago* seed coat mucilage and default parameters in ClustVis (Metsalu and Vilo, 2015). (**B**) Phylogenetic relationships between seven *Plantago* species as determined by ITS sequences, using *Veronica* species as outliers as per Ronsted et al., (2002).

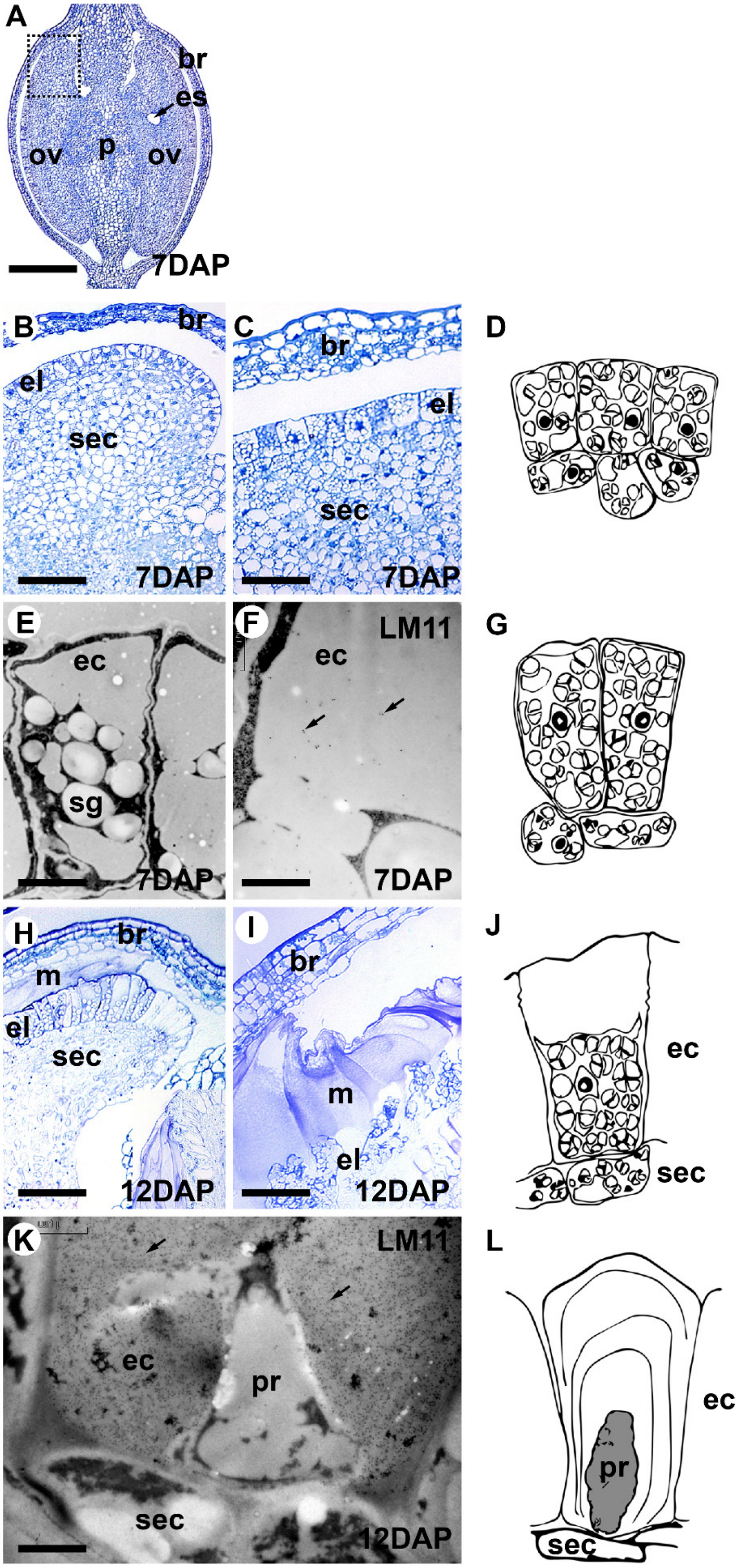

Fig. S4. Anatomical details of seed coat development and heteroxylan accumulation in *P. ovata*. (**A**) Toluidine blue stained thin section of a *P. ovata* fruit at 7DAP. (**B, C**) Magnified views of the boxed region indicated in A, showing the morphology of seed coat cells at 7DAP. (**D**) A schematic representation of the seed coat from a 0.8-1.0mm-long ovule at approximately 2-3DAP (adapted from Hyde, 1970). (**E**) Transmission electron microscopy of seed coat epidermal cells at 7DAP labelled with LM11 and a secondary gold-conjugated antibody. (**F**) Magnified view of E, showing sporadic LM11 labelling within the cell. Arrows indicate the gold particles. (**G**) A schematic representation of the seed coat from a 1.4-1.5mm-long ovule at approximately 4-5 DAP (adapted from Hyde, 1970) (**H, I**) Toluidine blue stained thin sections of *P. ovata* ovules at 12DAP. (**J**) A schematic representation of the seed coat from a 1.5-1.9mm-long ovule at approximately 7-8 DAP (adapted from Hyde, 1970). (**K**) Transmission electron microscopy of a seed coat epidermal cell labelled with LM11 and a secondary gold-conjugated antibody at 12DAP, showing extensive LM11 labelling within the cell. Arrows indicate the gold particles. (**L**) A schematic representation of the seed coat from a 2.3-2.7mm-long ovule at approximately 11-13 DAP (adapted from Hyde, 1970). ov, ovule, p, placenta, es, embryo sac, ca, carpel wall, el, epidermal layer, sec, sub-epidermal cells, ec, epidermal cell, sg, starch granule, m, mucilage, pr, protoplast. Bar in A = 70 µm, B, C = 20µm, E, F, K = 1µm

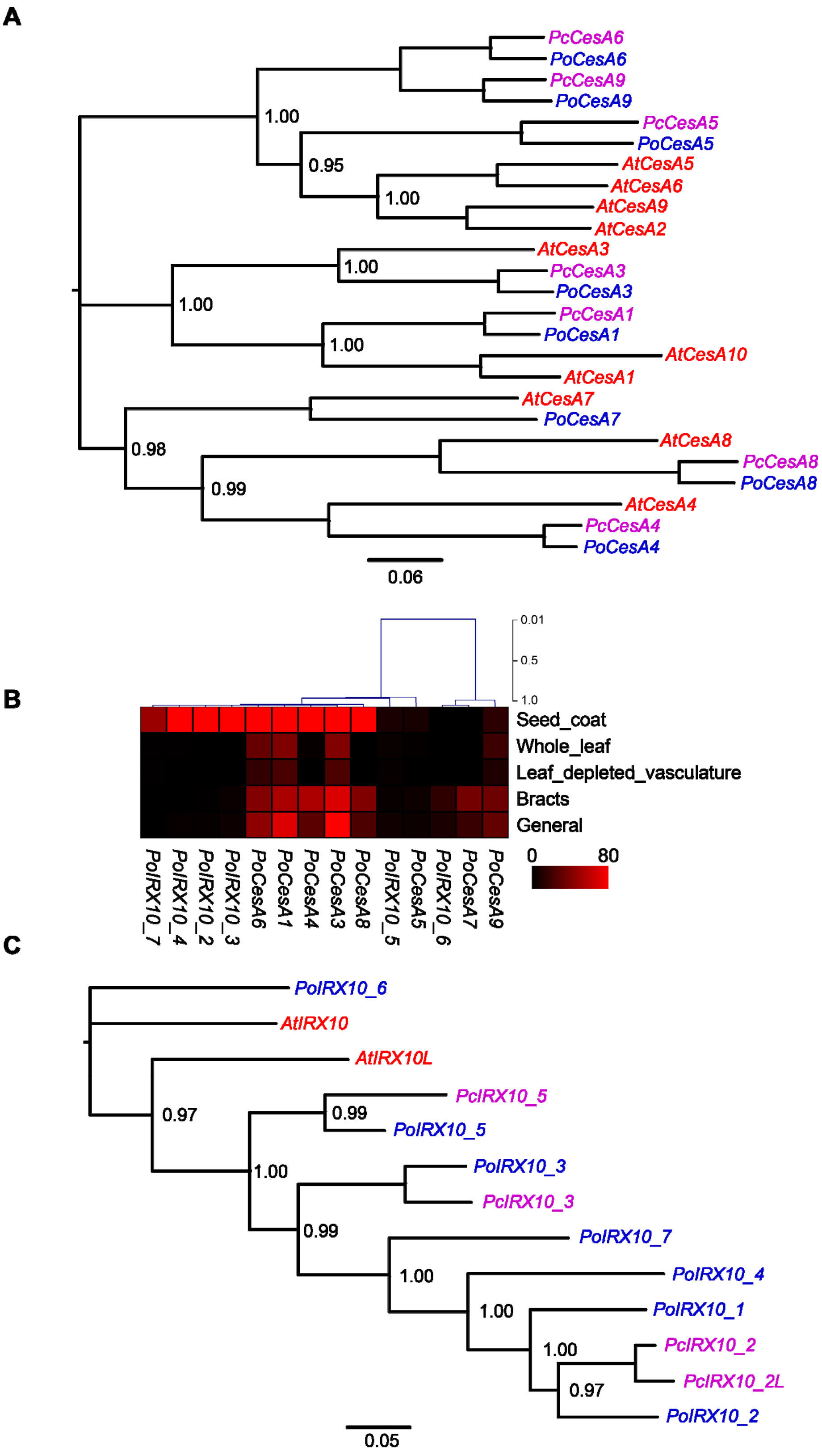

Fig. S5. Analysis of *Plantago* CesA and IRX10 sequences. (**A**) Phylogenetic relationships between CesA sequences from *Plantago ovata*, *Plantago cunninghamii* and *Arabidopsis thaliana*. (**B**) Heat map showing the relative transcript abundance (RPKM) of CesA and IRX10 sequences in different *P. ovata* tissues including seed coat (12-14DAP), whole leaf, whole leaf without vascular tissues, bracts and a general tissue pool. Samples were ordered using the hierarchical clustering function in MeV with default parameters but no sample ordering. The maximum cut-off was drastically reduced to 80 in order to visualise expression in tissues other than the seed coat. (**C**) Sequence relationships between IRX10 sequences from *Plantago ovata*, *Plantago cunninghamii* and *Arabidopsis thaliana*.

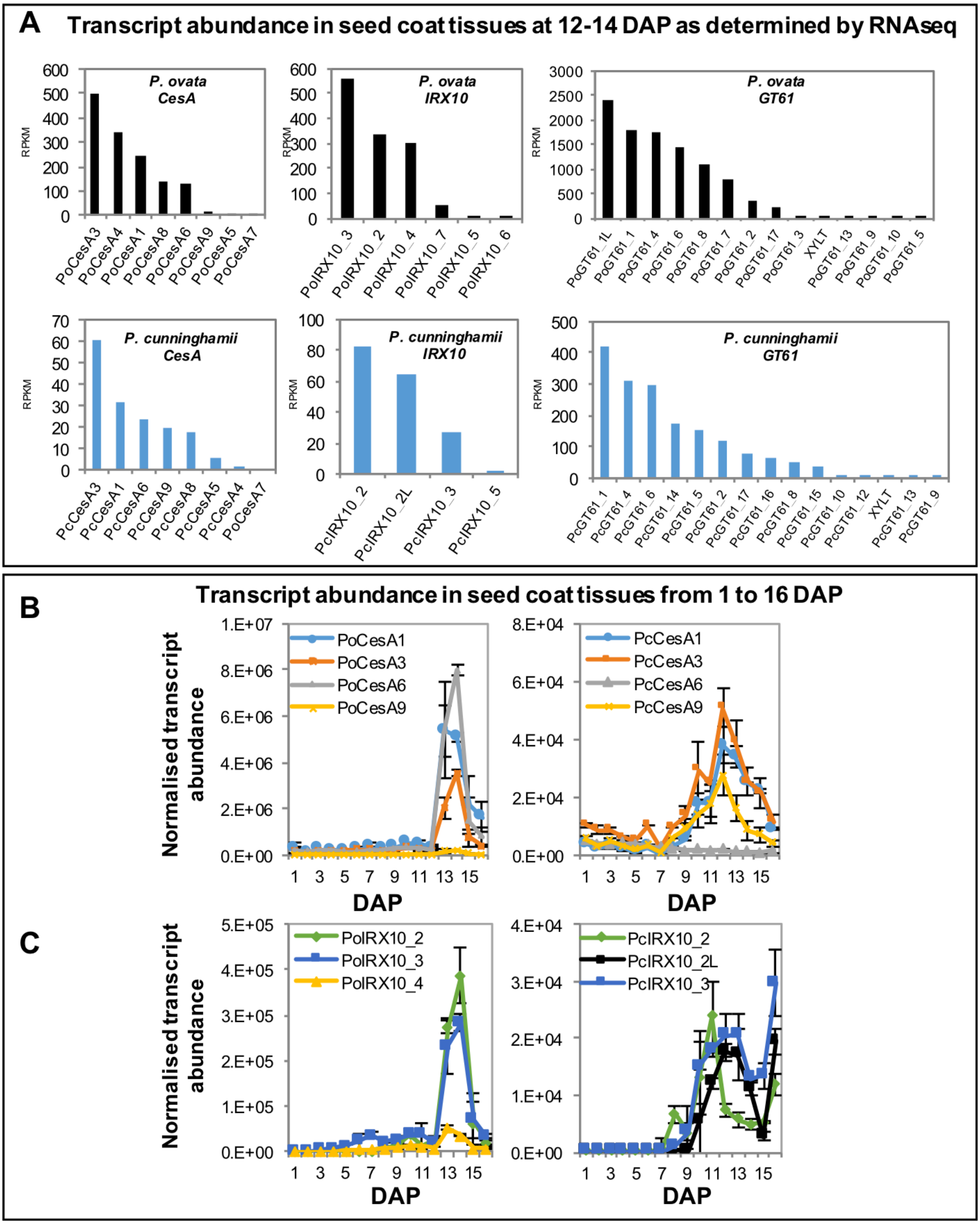

Fig. S6. Relative abundance of *Plantago ovata* and *Plantago cunninghamii* CesA, IRX10 and GT61 sequences in seed coat tissues. (**A**) Column graphs showing relative abundance of sequences in 12-14 DAP seed coat samples in terms of reads per kilobase per million reads (RPKM). Sequences were ranked according to the level of transcript. Each species was normalised independently. (**B**) Quantitative PCR profiles of 4 orthologous *CesA* genes in seed coat tissues from *P. ovata* and *P. cunninghamii*, 1 to 16 days after pollination (DAP). Error bars show standard deviation. (**C**) Quantitative PCR profiles of homologous *Plantago* IRX10_3 sequences during seed coat development in *P. ovata* and *P. cunninghamii.* In each plot for **B** and **C** the y-axis shows normalised expression values generated independently for each species. Error bars show standard deviation.

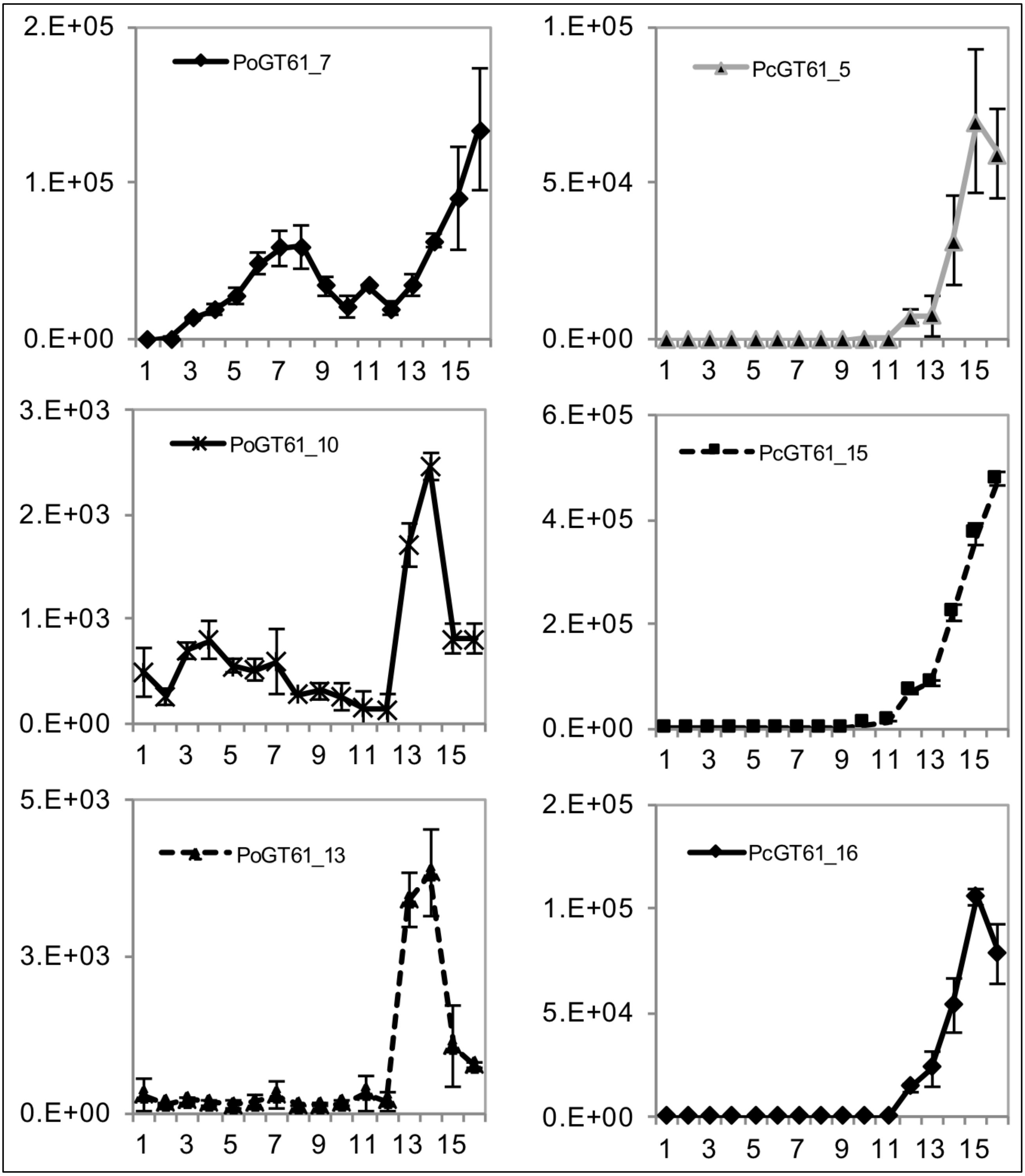

Fig. S7. Transcriptional profiles of selected *Plantago* GT61 family sequences during seed coat development in *P. ovata* and *P. cunninghamii.* In each plot the y-axis shows normalised expression values which were normalised independently for each species. Error bars show standard deviation.
